# Enhancer looping protein LDB1 modulates MYB expression in T-ALL cell lines in vitro by cooperating with master transcription factors

**DOI:** 10.1186/s13046-024-03199-1

**Published:** 2024-10-09

**Authors:** Yan Li, Zimu Zhang, Juanjuan Yu, Hongli Yin, Xinran Chu, Haibo Cao, Yanfang Tao, Yongping Zhang, Zhiheng Li, Shuiyan Wu, Yizhou Hu, Frank Zhu, Jizhao Gao, Xiaodong Wang, Bi Zhou, Wanyan Jiao, Yumeng Wu, Yang Yang, Yanling Chen, Ran Zhuo, Ying Yang, Fenli Zhang, Lei Shi, Yixin Hu, Jian Pan, Shaoyan Hu

**Affiliations:** 1 Children’s Hospital of Soochow University, Suzhou, China; 2 Department of Pediatrics, The Affiliated Hospital of Xuzhou Medical University, Xuzhou, China; 3 Institute of Pediatric Research, Children’s Hospital of Soochow University, SIP, No.92 Zhongnan Street, Suzhou, 215003 China; 4 Department of Hematology, Children’s Hospital of Soochow University, SIP, No.92 Zhongnan Street, Suzhou, Jiangsu China; 5 Department of Pediatric Surgery, The Affiliated Hospital of Yangzhou University, Yangzhou, China; 6 Department of Medical Biochemistry and Biophysics, Karolinska Institutet, 17165 Stockholm, Sweden; 7 Department of Internal Medicine, The Ohio State University, Columbus, 43210 USA; 8 Department of Pediatric, Suzhou Hospital of AnHui Medical University, Suzhou, 234000 China; 9 Department of Pediatric, Yancheng , Third People’ Hospital, YanCheng, 224000 China; 10 Department of Pediatric, The First Affiliated Hospital of Bengbu Medical College, Bengbu, 233004 China; 11 Clinical Medicine, Guizhou Medical University, Guiyang, 550000 China; 12 Department of Medicinal Chemistry, Jiangsu Key Laboratory of Drug Design and Optimization China Pharmaceutical University, Nanjing, 210009 China

**Keywords:** T-cell acute lymphoblastic leukemia,LDB1, CUT&Tag analysis, MYB, MBZ

## Abstract

**Background:**

Despite significant progress in the prognosis of pediatric T-cell acute lymphoblastic leukemia (T-ALL) in recent decades, a notable portion of children still confronts challenges such as treatment resistance and recurrence, leading to limited options and a poor prognosis. LIM domain-binding protein 1 (LDB1) has been confirmed to exert a crucial role in various physiological and pathological processes. In our research, we aim to elucidate the underlying function and mechanisms of LDB1 within the background of T-ALL.

**Methods:**

Employing short hairpin RNA (shRNA) techniques, we delineated the functional impact of LDB1 in T-ALL cell lines. Through the application of RNA-Seq, CUT&Tag, and immunoprecipitation assays, we scrutinized master transcription factors cooperating with LDB1 and identified downstream targets under LDB1 regulation.

**Results:**

LDB1 emerges as a critical transcription factor co-activator in cell lines derived from T-ALL. It primarily collaborates with master transcription factors (ERG, ETV6, IRF1) to cooperatively regulate the transcription of downstream target genes. Both in vitro and in vivo experiments affirm the essential fuction of LDB1 in the proliferation and survival of cell lines derived from T-ALL, with MYB identified as a significant downstream target of LDB1.

**Conclusions:**

To sum up, our research establishes the pivotal fuction of LDB1 in the tumorigenesis and progression of T-ALL cell lines. Mechanistic insights reveal that LDB1 cooperates with ERG, ETV6, and IRF1 to modulate the expression of downstream effector genes. Furthermore, LDB1 controls MYB through remote enhancer modulation, providing valuable mechanistic insights into its involvement in the progression of T-ALL.

**Supplementary Information:**

The online version contains supplementary material available at 10.1186/s13046-024-03199-1.

## Background

As a highly aggressive hematologic malignancy, T-cell acute lymphoblastic leukemia (T-ALL) comprises a small fraction of acute lymphoblastic leukemia cases, ranging from 10 to 15% in pediatric patients and increasing to as much as 25% among adults diagnosed with ALL [1, 2]. Children diagnosed with T-ALL have inferior overall survival (OS) when compared to those with B-cell ALL [2–5]. Despite treatment with chemotherapy regimens, steroids, and allogeneic transplantation, relapse poses a significant challenge in pediatric T-ALL cases [6]. Unfortunately, the outcomes for relapsed T-ALL remain poor, with a 5-year OS of 35% [7]. Patients facing refractory and relapsed T-ALL encounter limited therapeutic alternatives and a bleak prognosis [8]. Hence, exploring the underlying pathogenesis of T-ALL is crucial to improving therapeutic strategies and outcomes.

The precise control of gene expression programs is orchestrated by the coordinated interaction of cell lineage-specific key regulatory transcription factors and enhancers. These master TFs and enhancers serve as hubs that integrate chromatin states and TF binding, guaranteeing the elevated transcription of crucial genes linked to the identity, functionality, and endurance of cells [9]. In light of this, cells of leukemia and lymphoma sustain unusually elevated transcription rates for essential cancer-promoting genes. This elevated transcription is essential for expressing functional oncoproteins that sustain rapid tumor propagation and survival. T-ALL is distinguished by the activation of a series of important TFs, including TAL1, LYL1, HOX11 [10, 11]. These activated transcription factors, along with active signaling pathways, represent potential targets for therapeutic agents.

LDB1 is a ubiquitously expressed and evolutionarily conserved protein involved in chromatin looping, and it mediates the connection between enhancer and promoter regions across diverse cell types via its LIM and LIM homologous domains. Contemporary investigations revealed that the crucial roles of LDB1 in neurodevelopment [12], erythroid differentiation [13, 14], heart development [15, 16], regulation of liver cell gene expression [17], establishment and maintenance of pancreatic endocrine lineages [18] and in neural network-induced breast tumors [19]. Murine model experiments have reported that LDB1 plays a critical role in LMO2-driven thymic cell self-propagation, thymic cell radiosensitivity tolerance, and the transformation of pre-leukemic thymic cells into overt T-ALL [20]. A recent study investigating the functional mechanism of LDB1 in liver cells revealed that LDB1 primarily interacts cooperatively with liver master TFs GATA4, FOXA1, HNF4A, and TCF7. It was shown to bind to enhancers overlapping with these TFs at specific sites, organizing chromatin interactions, regulating the expression of metabolic genes in liver cells, and maintaining the essential metabolic functions of the liver [17]. Therefore, we hypothesize that there may be a synergistic interaction between LDB1 and master transcription factors in T-ALL cell lines. Experimental validation of this hypothesis could provide new therapeutic strategies for T-ALL treatment.

In our research, we observed that LDB1 knockdown affects the propagation and apoptosis of T-ALL cell lines. RNA sequencing (RNA-seq) revealed dysregulated expression of many key oncogenes after LDB1 loss in 6 T-CEM and J.gamma1. The CUT&Tag Assay showed that LDB1 primarily binds to enhancers overlapping with master TFs (ETV6, ERG, IRF1). LDB1 knockdown significantly decreased the expression of the T-ALL master oncogene MYB. Interestingly, we also discovered that LDB1 remotely regulates MYB expression through an unreported enhancer. Our study confirms the co-localization of LDB1 with master TFs in T-ALL cell lines, regulating MYB expression through the formation of transcription complexes, providing new therapeutic approaches for clinical therapy of T-ALL patients.

## Methods

### Cell culture

Jurkat, 6 T-CEM, CCRF, as well as J.gamma1 were obtained from the Chinese Academy of Sciences Cell Bank.SUP-T1and PF-382 were obtained from Zhejiang Meisen Cell Technology Co.,Ltd. The aforementioned cell lines were propagated in RPMI 1640 medium(22,400,089, Gibco, USA) supplemented with 10% fetal bovine serum(FBS)(Biological Industries, CT, USA). HEK293FT cells were propagated in full-strength DMEM medium (manufactured by Biological Industries; Sartorius AG), enriched with 10% FBS. Cells were cultured in a humidified incubator at 37℃ containing 5% CO2, and routinely tested for mycoplasma. Each cell line was authenticated through Short Tandem Repeat (STR) analysis.

### Lentiviral preparation and shRNA-mediated knockdown (KD) of LDB1

The shRNA was synthesized and its incorporation into the pLKO.1 vector were carried out by IGE Biotechnology LTD, China. HEK293FT cells of 10 cm dish size were transfected with 7.5 μg of purified plasmids together with packaging plasmids, 5.625 μg of and psPAX2 and1.875 μg of pMD2.G(MA,USA), Using PEI transfection reagent. The supernatant was harvested after incubating for 48 h at 37℃, subsequently filtered using a 0.45 μm-syringe filter, and then applied for infecting cells that were plated at a density of 2 × 10^**5**^ cells per well in a 6-well plate. After a 24-h incubation, the culture medium was refreshed with complete 1640 medium enriched with 1 µg/ml puromycin (ST551, Beyotime, China) to facilitate the selection of successfully infected cells. Three days following the selection process, we harvested the cells to achieve full knockdown of LDB1. Consequently, the infected cells were employed for various experiments. The sequences of shRNA utilized in this research are provided in Supplementary Table 1.

### Reverse transcription–Real-time quantitative PCR

Using the prescribed manufacturer's instructions, the collected cells were subjected to RNA extraction utilizing the TRIzol® reagent (Invitrogen, CA, USA). Subsequently, the extracted total RNA was reverse transcribed into cDNA using a high-capacity cDNA reverse transcription kit (Applied Biosystems, CA, USA). For PCR amplification, the reaction system was prepared with LightCycler®480 SYBR Green I Master mixture (Roche, Germany), and real-time PCR was performed on the LightCycler 480 system (Roche). The CT values corresponding to the amplification cycles of both the experimental samples and controls were standardized to the levels of GAPDH. Subsequently, the comparative gene expression levels were determined through the calculation of fold changes utilizing the 2^–ΔΔCT method. The quantification of a gene's relative expression level was derived by taking the mean of three separate measurements. The sequences of primers utilized within the research are provided in Supplementary Table 2.

### Western blotting analysis

Cells harvested underwent a cleansing step with phosphate-buffered saline and were subsequently subjected to lysis with RIPA buffer (Beyotime, China). Protein samples, quantitatively matched, underwent separation via SDS-PAGE, following which they were applied to polyvinylidene difluoride (PVDF) membranes (CST, USA). After blocked with a 5% non-fat milk solution in TBST and incubated with primary antibodies while being agitated at a temperature of 4℃ throughout the night, the PVDF membrane was incubated with a secondary antibody at room temperature. Then the membrane was coated with an ECL detection solution (Millipore, USA) to visualize the protein bands. The chemiluminescence emitted was captured with an AI600 gel documentation system (GE, USA) for image analysis. The antibodies employed in this research are documented in Supplementary Table 3.

### CCK-8 and soft agar colony formation assay

Subsequent to puromycin-mediated selection, the infected cells were seeded at a concentration of 5,000 cells per well into 96-well plates. 20 μl of CCK-8 solution(APExBIO, K1018-1, USA) was dispensed to every individual well containing 200 μl of 1640 medium with infected cells on the 1st/3rd/5th days. The plates underwent incubation at a temperature of 37℃ for 2 h, post which a spectrophotometer (Thermo, USA) was utilized to measure the absorbance at a wavelength of 450 nm. Cells from both the shRNA negative control and shRNA targeting LDB1 groups were seeded into a soft agar culture medium. Following a period of approximately two weeks of incubation, the cells underwent a staining process using Giemsa stain (Product code C0131 from Beyotime, China), subsequently enabling the counting of cell colonies formed.

### EdU assays

For 5-ethynyl-2-deoxyuridine Edu assays, the proliferation ability of cells was evaluated using a BeyoClick™ EdU Cell Proliferation Kit with Alexa Fluor 594 (Cat#: C0078S; Beyotime).The procedure was performed according to the manufacturer’s instructions. Prepare control and LDB1 knockdown cells in advance. Subsequently, collect control and experimental group cells after 5 days of puromycin selection for EdU staining. After EdU staining, analyze the collected control and experimental group cell suspensions using flow cytometry.

### Colony formation assay

Add 20 mL of Iscove's Modified Dulbecco's Medium (Catalog #36,150, Stem Cell Technologies, Canada) to 80 mL of MethoCult™ H4230(Catalog #04230, Stem Cell Technologies, Canada) methylcellulose medium and mix thoroughly by vigorously shaking the bottle for 1–2 min. Then, let the mixed MethoCult medium sit at room temperature for 15–20 min to allow bubbles to rise to the top. Aliquot the medium into sterile distribution tubes, with 3 mL in each tube, for later use. Two days after puromycin selection, both transfected control cells and LDB1 knockdown cells were collected. Dilute the cells from both control and experimental groups in IMDM medium containing 2% FBS to achieve a 10X final concentration. Add 0.3 mL of the cell suspension to each aliquoted 3 mL MethoCult medium and vortex for 5 min. Allow bubbles to rise to the top, then plate the cell suspension into 6-well plates at a final concentration of 10,000 cells/mL. To ensure adequate humidity, add sterile double-distilled water to the adjacent wells of the cell culture plates. Incubate the cells in a humidified incubator at 37 °C with 5% CO_2_. Colony formation was observed 10 days post-plating.

### Cell apoptosis assay

The infected cells after puromycin selection were initially rinsed with chilled 1 × phosphate-buffered saline (PBS) first. Subsequently, the samples were resuspended in 1 × Annexin V binding buffer and labeled with FITC-Annexin V antibody as well as propidium iodide (PI) following the guidelines provided with the FITC-Annexin V Apoptosis Detection Kit (catalog no. 556547, BD Biosciences, USA). Apoptotic cells were quantified utilizing the Gallios™ Flow Cytometer from Beckman (Beckman Coulter, Krefeld, Germany).

### Cell cycle assay

Following the manufacturer’s protocol, samples used for cell cycle assays were fixed overnight with pre-cooled 75% alcohol at 4 °C overnight. On the following day, the cells were washed with cold PBS and resuspended with PI dye and RNase A (cat. No. 550825; BD Pharmingen™, San Diego, CA, USA) and then incubated at room temperature for 15 min. The cell cycle was analyzed using Beckman Gallios™ flow cytometry (Beckman, Krefeld, Germany).

### RNA‑seq analysis and data processing

RNA extraction, library preparation, transcriptome sequencing (on the Illumina NovaSeq 6000 platform), and raw data filtering were conducted by Novogene Bioinformatics Technology Co., Ltd. (Beijing, China). Alignment of the 150 bp paired-end sequences was executed against the hg38 (Ensembl) human reference genome using HISAT2 (version 2.2.0). The reconstruction and quantification of the transcriptome were carried out employing StringTie (version 2.1.2). The R/Bioconductor package DESeq2 facilitated the identification of genes exhibiting differential expression, adhering to a threshold of an adjusted P-value < 0.05 and an absolute fold change (FC) >| 1|. Gene Set Enrichment Analysis (GSEA) was carried out with the R/Bioconductor package clusterProfiler, leveraging the wikipathways collection (2023.1 release) sourced from the Molecular Signatures Database (MSigDB).

### In vivo experiments

Female NSG mice aged 4–6 weeks were purchased from Shanghai Nanfang Model Biotechnology Inc. 6 T-CEM cells expressing firefly luciferase were prepared beforehand and transfected with viral particles carrying either sh-NC or sh-LDB1. Samples were collected on the third day following transfection. The NSG mice were divided into two groups using random assignment, with each mouse receiving an intravenous tail vein injection of a PBS-based cell suspension, consisting of 2 × 10^^6^ cells. D-luciferin sodium salt (GOLDBIO, USA) was administered intraperitoneally to tumor-bearing mice on D21/28/35 days after tail vein injection respectively. Then, using a small animal live imaging scanner from Berthold, Germany, quantification of the maximal standard mean radiance emitted by mouse tumors was performed. Liver, spleen, and bone tissues were harvested from both the experimental and control groups for the purposes of performing H&E staining and immunohistochemistry.

### Cleavage under targets and tagmentation (CUT&Tag)

 Assay

The CUT&Tag experiment was conducted on human 6 T-CEM, Molt4, Loucy and J.gamma1 cells utilizing the Hyperactive Universal CUT&Tag Assay Kit (TD903-01, Vazyme) following the guidelines provided by the manufacturer. 2 × 10^6^ cells were incubated with cold Nuclear Extract Buffer for 10 min. After centrifugation, the cell precipitate was resuspended using 500 μl of a 0.1% formaldehyde solution in the sample tube. The sample was then incubated at room temperature for 2 min before the cross-linking reaction was Terminated by adding 2.5 M glycine. The cell precipitate obtained after centrifugation was collected and was resuspended using a 100 μl wash buffer. Subsequently activated ConA Beads were added for further incubation, and then mixed with primary antibodies, which include: LDB1, ERG, IRF1, ETS1, ETV6, and IgG mentioned above. The samples, along with the aboving antibodies, were incubated overnight at 4 °C. The following day, 50 μl of secondary antibodies diluted at a Proportion of 1:100 were added to each sample. The mixtures were then subjected to rotational incubation at room temperature for 1 h. The samples were next fragmented and DNA were extracted. Novogene carried out the sequencing of all CUT&Tag libraries, employing the PE150 protocol on the Illumina NovaSeq 6000 sequencing platform(Novogene, Beijing, China).

### Immunoprecipitation

6 T-CEM and J.gamma1 cells (transfected with Ldb1-Flag overexpression constructs) were lysed using a buffer including NP-40 (P0013F, Beyotime, China) along with a protease inhibitor. Following a 30-min incubation at 4 °C in a refrigerated centrifuge, the lysate was centrifuged to obtain the supernatant for collection. Subsequently, A total of 50 μl lysate was mixed with 5 × loading buffer and boiled at 100℃ for 10 min to denature the proteins, serving as the input group. The protein A/G beads (B23202, Bimake, China) were mixed with the other two parts of the supernatant, while LDB1 antibody or IgG Antibodies were used in the respective mixtures. The mixtures were subsequently subjected to overnight rotation at 4 °C. The next day, the samples were subjected to centrifugation at 8200 g to remove the supernatant. Subsequently, the pellets were washed five times with pre-chilled TBST to remove the remaining supernatant. The pellets were then re-suspended in 2 × loading buffer and heated to induce denaturation, for subsequent use in Western blot analysis.

### Statistical analysis

Three separate experiments were executed. All statistical analyses were performed using GraphPad PRISM 8.0.2 software. The two groups were compared by double-tailed unpaired Student's t test for data analysis. Statistical significance was ascribed to P values less than 0.05 as follows: *p < 0.05, **p < 0.01, ***p < 0.001, ****p < 0.0001. Survival curve analysis was compared by Log-Rank test, and *P* < 0.05 indicate statistical significance.

## Results

### Upregulation of LDB1 in T-ALL patients and its correlation with poor clinical characteristics

**Fig. 1:**
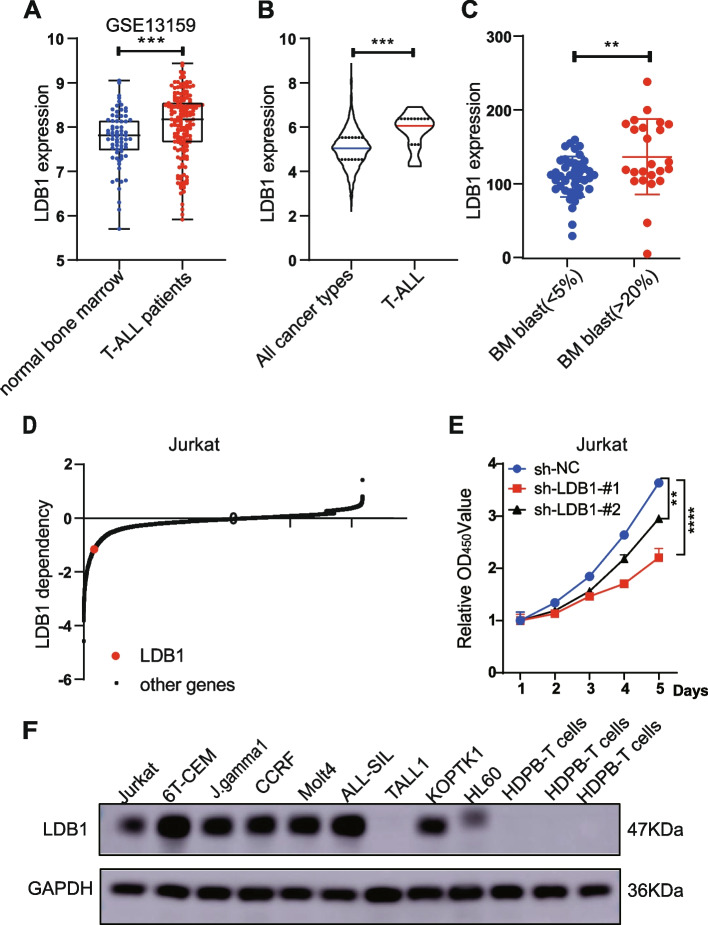
Upregulation of LDB1 in T-ALL patients and its correlation with poor clinical characteristics. **A** Data from the GEO dataset GSE13159 revealed a significantly higher mRNA expression of LDB1 in T-ALL patients compared to bone marrow samples from healthy donors. **B** Analysis of the Cancer Cell Line Encyclopedia (CCLE) database revealed an overexpression of LDB1 in T-ALL cell lines compared to other cancer types. **C** Our center's sequencing data of T-ALL patients demonstrated a statistically higher expression of the LDB1 gene in patients with a high bone marrow tumor burden. **D** CRISPR screen data indicated the dependence of T-ALL cell line Jurkat on LDB1. **E** CCK-8 assay detected the proliferation rate of Jurkat cell. **F** Western blot assay for LDB1 protein expression in acute leukemia cell lines and Healthy Donor T Cells

To elucidate the role of LDB1 in the context of T-ALL, we initially conducted an analysis of the gene expression profiles of LDB1 using publicly accessible datasets. Analysis of the GEO dataset GSE13159 unveiled a notably elevated mRNA expression of LDB1 in T-ALL patients compared to bone marrow samples from healthy donors (Fig. 1A). Furthermore, analysis of the Cancer Cell Line Encyclopedia (CCLE) database revealed an overexpression of LDB1 in T-ALL cell lines compared to other cancer types (Fig. 1B). Consistent with these findings, our center's sequencing data of T-ALL patients, categorized based on bone marrow status post-induction therapy demonstrated a statistically higher expression of the LDB1 gene in patients with a high bone marrow tumor burden (Fig. 1C). The Table 1 presents the clinical features or traits exhibited by the patients. Additionally, CRISPR screen data indicated the dependence of T-ALL cell line Jurkat on LDB1 (Fig. 1D, E and Supplementary Table 4). In comparison to healthy donors' peripheral T-lymphocytes, western blot analysis revealed elevated levels of LDB1 in T-ALL cell lines (Fig. 1F). Collectively, the findings of our research highlight the particular upregulation of the LDB1 gene in T-ALL, and an increased level of LDB1 is linked to a negative outlook, underscoring the significant pathogenic role of LDB1 in T-ALL patients.

**Table 1:** Clinical characteristics of Pediatric T-ALL patients

	Pediatric T-ALL patients (*n*=84)
Gender, n(%)	
Male	60(71.4)
Female	24(28.6)
Age at diagnosis, years	7.7(1.2-13.3)
Initial WBC(×10^9^)	
<100	32(38.1)
>100	52(61.9)
Hemoglobin, g/L, median, range	102(37-159)
Platelet, (×10^9^), median, range	77(10-263)
Genetic subtypes, n(%)	
SIL/TAL1	24(28.6)
MLLr	5(5.9)
Hox11	7(8.3)
TLX1/TCRα AHI1/MYB	1(1.2)
PTEN/PCSK5	1(1.2)
Karyotype	
normal	47(55.9)
t(11,14)(p13,q11)	5(5.9)
Other structural abnormal	10(11.9)
Numerical abnormal	6(7.1)
Failure or missing	16(15.2)
Prednisone Response, n(%)	
Poor	46(54.8)
Good	38(45.2)
D15 BM Blast, n(%)	
<5%	48(57.1)
>20%	36(42.9)
Risk group, n(%)	
Intermediate risk	57(67.8)
High risk	27(32.2)
Survival status	
Survival	58(69.0)
death	26(31.0)

### Altered LDB1 xpression affects the proliferation of T-ALL cells

**Fig. 2:**
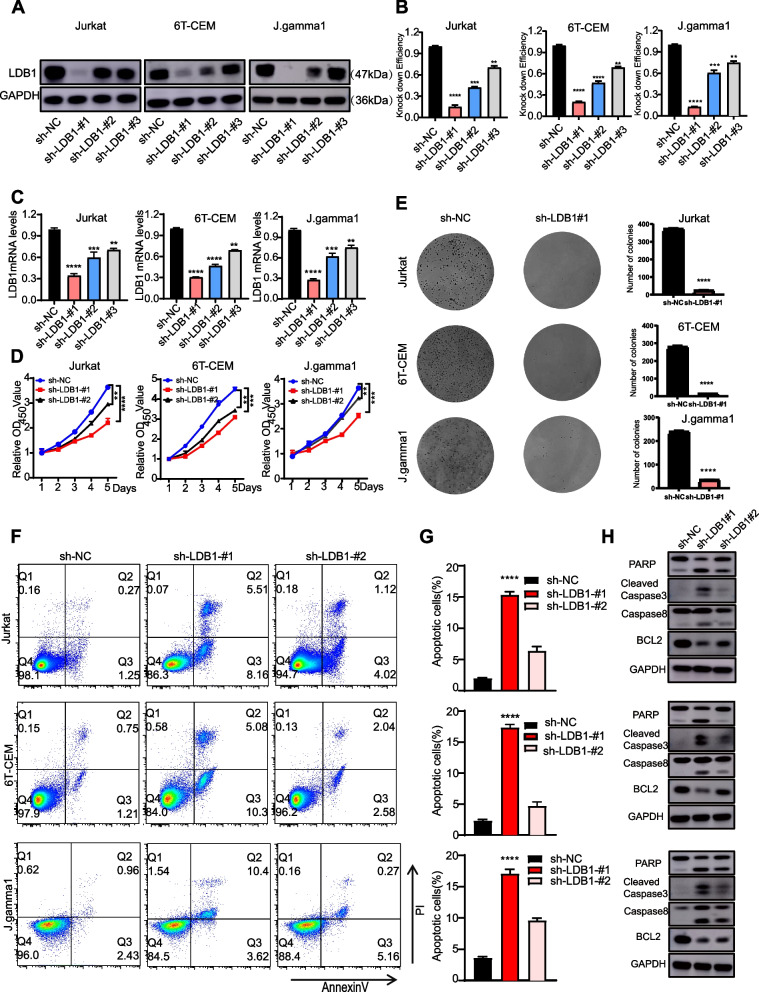
Altered LDB1 expression affects the proliferation of T-ALL cells. **A** The knockdown levels of LDB1 in cells was verified by WB. **B** Western blotting analysis showed that LDB1 and GAPDH protein pression in Jurkat, 6 T-CEM and J.gamma1 cells after LDB1 knockdown. **C** RT-PCR analysis showed that LDB1 and GAPDH protein pression in Jurkat, 6 T-CEM and J.gamma1 cells after LDB1 knockdown. **D** CCK-8 assay detected the proliferation rate of Jurkat, 6 T-CEM and J.gamma1 cells after LDB1 knockdown. **E** Colony-forming assay for Jurkat, 6 T-CEM and J.gamma1 cells infected with sh-NC or sh-LDB1#1. **F** Flow cytometry using Annexin V staining showed that knockdown of LDB1 increased the apoptotic rates of Jurkat, 6 T-CEM and J.gamma1 cell lines. **G** Knockdown of LDB1 increased the Annexin V^+^375 fraction and promoted apoptosis in Jurkat, 6 T-CEM and J.gamma1 cell lines. **H** Western blotting analysis showed that the PARP, Cleaved Caspase3 and Cleaved Caspase8 were upregulated in T-ALL cells after LDB1 knockdown, while BCL2 was downregulated

To elucidate the role of LDB1 in T-ALL, we conducted transfection experiments using LDB1-targeting short hairpin RNAs(sh-LDB1#1, sh-LDB2#1and sh-LDB1#3), along with control short hairpin RNAs (sh-NC) in Jurkat, 6 T-CEM and J.gamma1. By conducting western blot and reverse transcription polymerase chain reaction (RT-PCR) analyses, we detected a substantial reduction in the expression of LDB1 at both the protein and RNA levels in cells transfected with LDB1 shRNAs in comparison to the control group (Fig. 2A, B, and C). Additionally, we further assessed the impact of LDB1 on cell proliferation using Jurkat, 6 T-CEM and J.gamma1. The CCK8 results showed that cell proliferation was significantly inhibited in the LDB1-knockdown group compared to the control group (Fig. 2D). Furthermore, the results from the white slice assay demonstrated that the knockdown of LDB1 substantially impeded the proliferation of T-ALL cells, when compared to the control ( Fig. S1A). Also EdU-594 assays showed a significant inhibition of DNA synthesis in the LDB1 knockdown group compared to the control group in 6 T-CEM and Jurkat cells five days after virus transfection, indicating that cell proliferation was suppressed in the LDB1 knockdown group ( Fig. S1B). Simultaneously, we verified that knockdown of LDB1 induces cell cycle arrest in the G0/G1 phase ( Fig. S2A, B). Soft agar assays demonstrate a considerable decrease in the colony-forming ability of T-ALL cells upon LDB1 knockdown (Fig. 2E). Following LDB1 knockdown in Jurkat, 6 T-CEM, and J.gamma1 cell lines five days after virus transfection, flow cytometry analysis revealed an elevated proportion of apoptotic cells (Fig. 2F and D). We observed similar apoptotic results in the newly acquired T-ALL cell lines, PF-382 and SUP1 ( Fig. S2C, D). Consistent with these findings, western blot analysis uncovered a reduction in the expression of Bcl2, along with the presence of cleaved bands of PARP, cleaved caspase-3 and caspase-8 five days after virus transfection (Fig. 2H). Taken together, these findings strongly indicate that the abnormal expression of LDB1 exerts a pivotal role in determining the survival of T-ALL cells.

### The anti-tumor impact of lowering LDB1 levels in a mouse model of leukemia

**Fig. 3:**
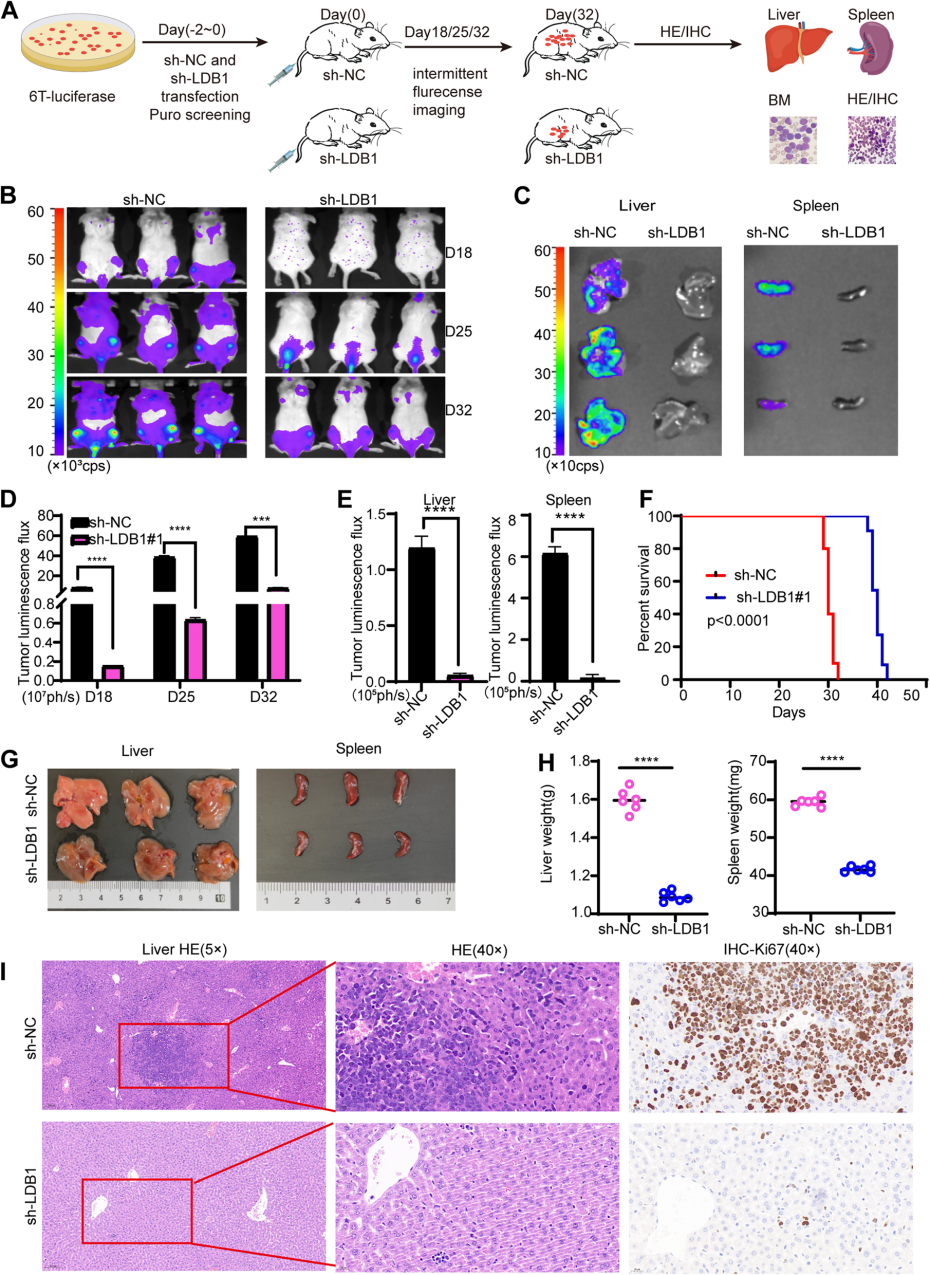
Anti-tumor effect of LDB1 knockdown in leukemia mouse model. **A** Schematic diagram of the vivo experimental design. **B** Relevant bioluminescence imaging of D18, D25 and D32 days in LDB1 knockdown group and control group. **C** Biological image of Avg radiance uptake by liver and spleen tissue between two groups. **D** Histogram shows the bioluminescence signal values for both groups of mice at different time points. **E** Histogram shows the bioluminescence signal values of liver, and spleen. **F** Mice in the LDB1 knockdown group exhibited prolonged survival time compared to the control group. **G** Different sizes and weights of liver and spleen, from sh-NC or sh-Ldb1 mices. **H** The liver and spleen weights show statistical differences between the two groups. **I** Representative images of HE staining and IHC staining of mice livers

To further investigate the influence of LDB1 on the proliferation of T-ALL cells in mice, we initially labeled 6 T-CEM cells with luciferase and then intravenously injected LDB1-targeted shRNA and sh-NC cells into NSG mice via the tail vein (Fig. 3A). As depicted in Fig. 3B, the fluorescence signal intensity of mice in the LDB1 knockdown group demonstrated a substantial reduction in comparison to the control group. Additionally, visualization of liver and spleen samples of mice using fluorescence imaging indicated a substantial reduction in tumor burden within the LDB1 knockdown group when compared to the control group (Fig. 3C). In comparison to the control group, the tumor luminous flux histogram of LDB1 knockdown group exhibited a significant decrease (Fig. 3D, and E). Moreover, the LDB1 knockdown mice exhibited a substantial improvement in median survival, presenting a significant advantage over the control group (Fig. 3F). Furthermore, mice in the sh-NC group displayed larger liver and spleen sizes compared to those in the Ldb1 knockdown group (Fig. 3G). The liver and spleen weights show statistical differences between the two groups (Fig. 3H). H&E staining and hCD45 flow cytometry analysis of the mouse liver, spleen, and bone marrow demonstrated a notable reduction in tumor cells in the LDB1 knockdown group in comparison to the control group (Fig. 3I and Fig. S3A-D). According to the results from immunohistochemical (IHC) staining of Ki67 in liver and spleen tissues, it was verified that the knockdown of LDB1 exhibits a suppressive effect on tumor development (Fig. 3I and Fig. S3A). In conclusion, the outcomes of experiments performed in in vivo and in vitro are consistent, collectively demonstrating that LDB1 downregulation demonstrates a tumor-suppressive effect in cases of leukemia.

### Transcriptome analyses show dysregulated self-renewal in LDB1-knockdown T-ALL cells

**Fig. 4:**
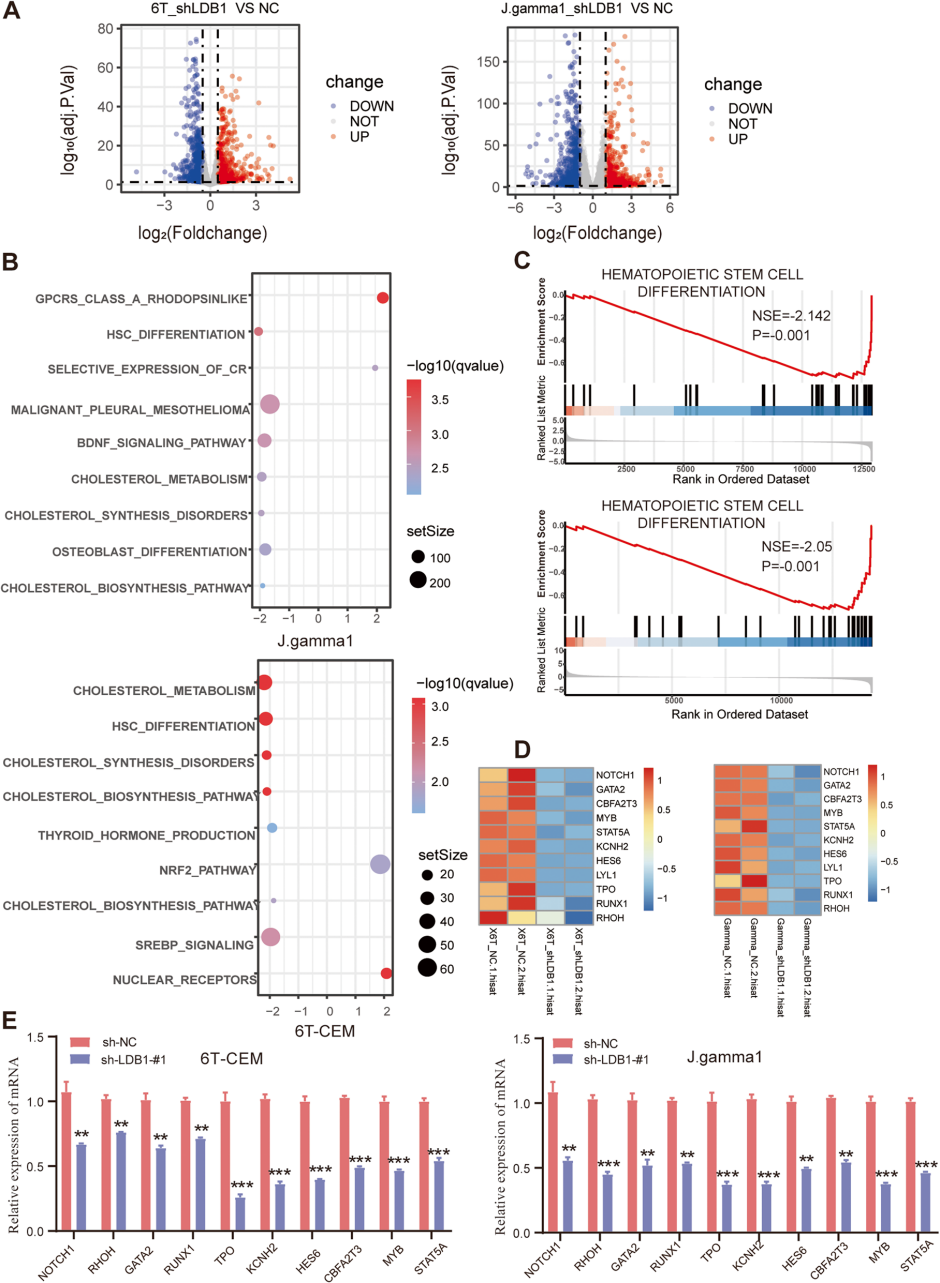
Transcriptome analyses show dysregulated self-renewal in LDB1-knockdown T-ALL cell. **A** Volcano plot analysis revealed differentially expressed genes obtained from RNA-seq data between the LDB1 knockdown and the control groups. Red and blue indicate upregulated and downregulated genes, respectively. **B** KEGG pathway enrichment analysis. **C** GSEA plots demonstrated gene enrichment in hematopoietic stem cell differentiation signaling pathways in T-ALL cells treated with LDB1 knockdown. **D** Heatmap view displayed the expression levels of genes sustaining the hematopoietic stem cell differentiation in 6 T-CEM and J.gamma1 cells treated with LDB1 knockdown. **E** the qPCR analysis showed that the expression levels of genes sustaining the hematopoietic stem cell differentiation, such as NOTCH1, MYB, and RUNX1, were decreased

To explore the intrinsic molecular mechanisms through which LDB1 exerts in T-cell acute lymphoblastic leukemia (T-ALL), we conducted RNA-seq analysis and revealed that 1151 differentially expressed genes (DEGs) were found in LDB1-knockdown J.gamma1 cells whereas 1941 DEGs were found in LDB1-knockdown 6 T-CEM cells in contrast to the control group (Fig. 4A, Supplementary Table 5, 6). Gene Set Enrichment Analysis (GSEA) with wikipathways sets for LDB1-knockdown cell lines and the control group showed significant enrichment for pathways involved in hematopoietic stem cell differentiation (Fig. 4B and C, Supplementary Table 7, 8). Consistently, the qPCR analysis with cells of four days and six days post-viral transduction showed that gene expression levels sustaining the hematopoietic stem cell differentiation, such as NOTCH1, MYB, and RUNX1, were decreased (Fig. 4D and E, Figure. S4A-C). To further assess dysregulated self-renewal in LDB1-knockdown T-ALL cell, we conducted a CFU assay on Jurkat and PF-382 cell lines. The results showed that compared to the control group, the colony-forming ability of cells was significantly decreased in the LDB1 knockdown group (Figure. S5A). Additionally, we performed stem cell marker CD34 analysis on Jurkat and PF-382 cell lines using flow cytometry. The results revealed that the stem cell marker CD34 was significantly reduced in the LDB1 knockdown group compared to the control group (Figure. S5B).

### LDB1 cooperates with master transcription factors to control gene transcription

**Fig. 5:**
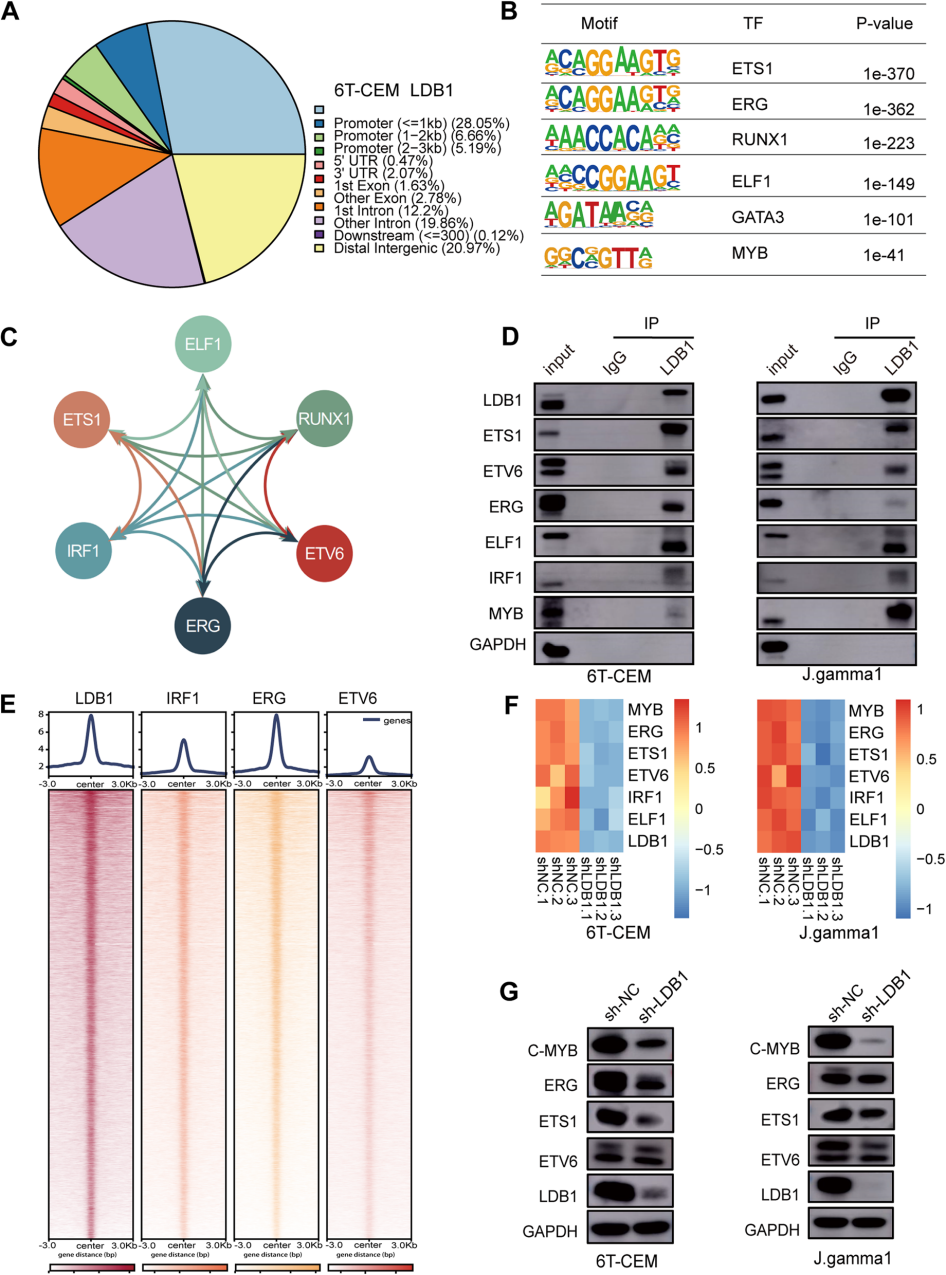
Cooperation between LDB1 and master transcription factors to regulate genes transcription. **A** Distribution of LDB1 binding to genomic regions in the 6 T-CEM cells, as assessed by CUT&Tag. The pie chart showing the peak annotation of LDB1. **B** Motif analysis of LDB1 binding sites in 6 T-CEM cells using Homer (22).** C** Potential core transcriptional regulatory circuitry were constructed by analyzing H3K27ac ChIP data from T-ALL patient samples. **D** LDB1 immunoprecipitation successfully captured GATA3, ETS1, ERG, RUNX1, ELF1, MYB, as well as IRF1.** E** Co-localization of LDB1 with IRF1, ERG, and ETV6 on chromatin were observed. **F.** RT-PCR analysis showed a significant reduction in the expression of MYB, ERG, ETS1, and ETV6. **G** A significant reduction in the expression of MYB, ERG, ETS1, and ETV6 was verified by WB

To identify the binding profile of LDB1 in 6 T-CEM cells, we conducted LDB1 CUT&Tag analysis, revealing 26,479 peaks. Approximately 40% of these peaks were located at promoters, while about 60% were situated in intergenic or intronic regions associated with enhancers (Fig. 5A, Supplementary Table 9). LDB1 depends on interacting proteins to associate with chromatin and modulate the transcription of distant genes. An analysis of the LDB1 binding sites using Homer revealed that LDB1 peaks showed a high abundance of the canonical motif. Motif analysis of LDB1 binding sites in 6 T-CEM cells using Homer indicated that LDB1 peaks were enriched for the canonical motif of several master transcription factors driving T-ALL progression, such as ETS1, RUNX1, GATA3, and MYB (Fig. 5B). The interconnection of master transcription factors and the core transcriptional regulatory circuitry is crucial for maintaining cell identity and status. By analyzing H3K27ac ChIP data from T-ALL patient samples, we constructed potential core transcriptional regulatory circuitry (Fig. 5C) and validated them through CUT&Tag experiments (Figure. S6A-C and Fig. S7A-B, Supplementary Table 10, 11, 12). We observed co-localization of LDB1 with IRF1, ERG, and ETV6 on chromatin, and found mutual transcriptional activation among IRF1, ERG, and ETV6 (Fig. 5E and Fig. S8A-D). Additionally, LDB1 immunoprecipitation successfully captured ETV6, ETS1, ERG, ELF1,IRF1, as well as MYB (Fig. 5D). Silencing LDB1 using shRNA in 6 T-CEM and J.gamma1 cell lines led to a noticeable decrease in the expression of MYB, ERG, ETS1, and ETV6 at both RNA and protein levels (Fig. 5F and 5G), indicating the crucial role of LDB1 in participating in the maintenance of the core transcriptional regulatory circuitry.

### MYB serves as a crucial downstream molecule of LDB1 complex

**Fig. 6:**
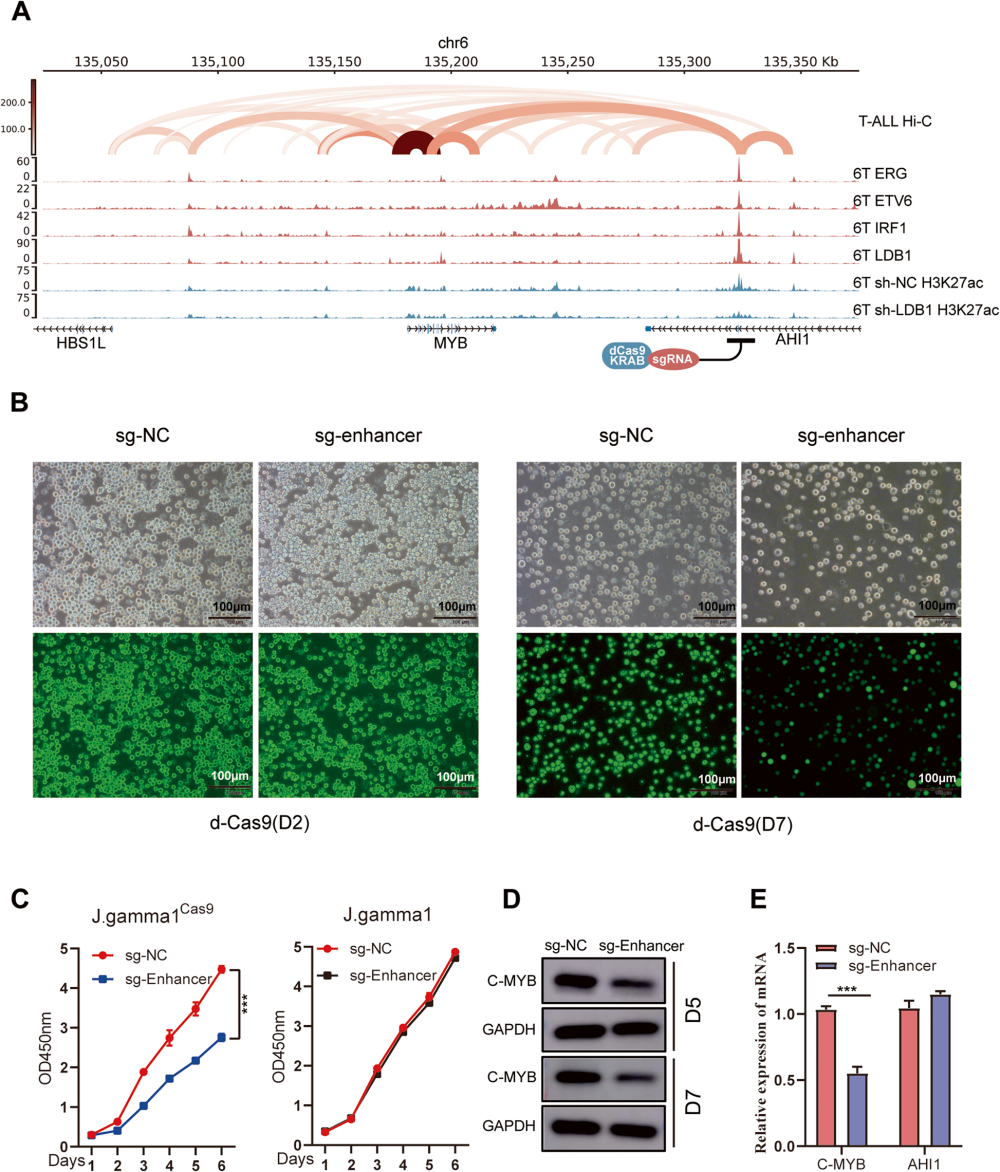
MYB serves as a crucial downstream molecule of LDB1 complex. **A** IGV visual analysis highlights that the binding profile of LDB1 complex, along with T-ALL Hi-C data, was analyzed and reveal that there is binding at distal enhancers in the Myb-AHI1 intergenic region. Through H3K27ac CUT&Tag experiments, a significant reduction in H3K27ac enrichment was observed in this enhancer region after LDB1 knockdown.** B** Results from the white slice experiment show that knocking out the enhancer sequence remarkably decreased the J.gamma1cas9 cells proliferation in comparison to the control group.** C** Knocking out the enhancer sequence in Jgamma1cas9 cells impaired cell proliferation while knocking out the enhancer sequence in J.gamma1 cells had no remarkable impact on cell proliferation. **D** Knockout of the identified enhancer sequence resulted in diminished c-Myb protein expression levels. **E** RT-PCR analysis showed that knockout of the identified enhancer sequence resulted in diminished c-Myb RNA expression levels, while there was no statistically significant difference in the expression levels of the AHI1 gene

The binding profile of LDB1 complex, along with T-ALL Hi-C data, was analyzed and reveal that there is binding at distal enhancers in the MYB-AHI1 intergenic region, suggesting potential regulation of MYB transcription through this enhancer region. The H3K27ac histone modification mark indicates the activity of transcriptional gene promoters and enhancers. Through H3K27ac CUT&Tag experiments, a significant reduction in H3K27ac enrichment was observed in this enhancer region after LDB1 knockdown (Fig. 6A). Additionally, the binding profile analysis of the LDB1 complex in other T-ALL cell lines such as Loucy, Molt-4, and J.gamma1 similarly revealed binding at distal enhancers in the MYB-AHI1 intergenic region (Figure.S10A, B,Supplementary Table 15, 16,17). Furthermore, we employed the CRISPR interference system, where single guide RNAs (sgRNAs) (Supplementary Table 14) guide the dCas9/KRAB complex to suppress this enhancer region. Subsequently, a significant decrease in both MYB mRNA and protein levels was observed (Fig. 6 D and E), leading to a pronounced inhibition of 6 T cell proliferation (Fig. 6B and C**)**.

### MBZ demonstrates anticancer effects both in vivo and in vitro

**Fig. 7:**
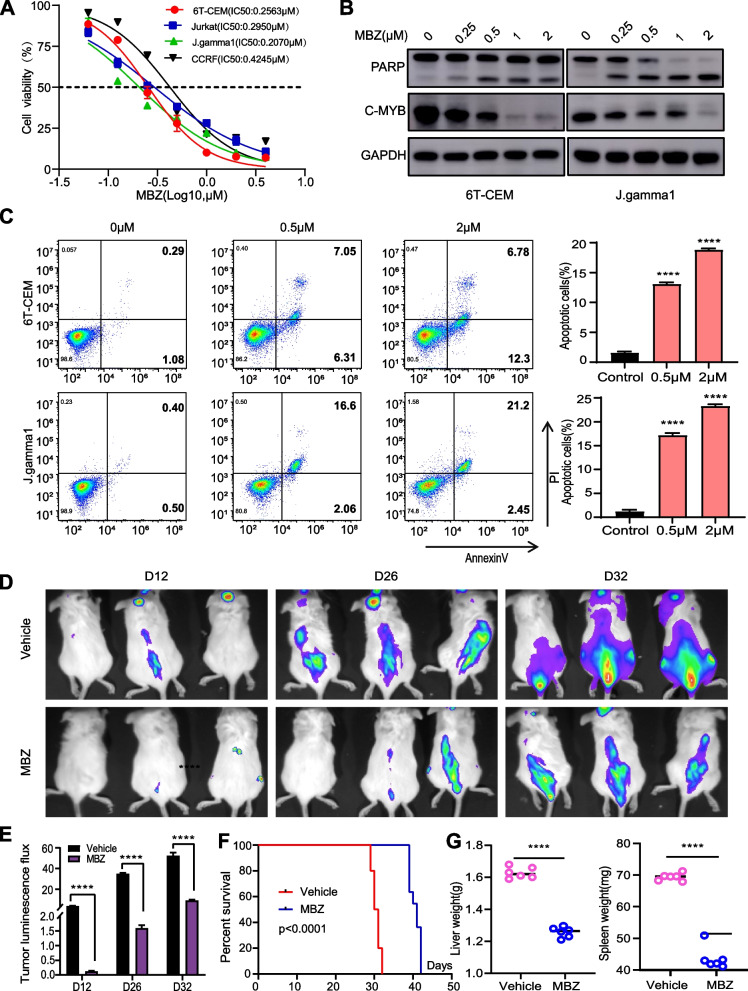
MBZ demonstrates anticancer effects both in vivo and in vitro. **A** CCK-8 assays detected the effects of MBZ. Robust inhibition of tumor cell growth in all four T-ALL cell lines. **B** Western blotting analysis showed that MYB, PARP and GAPDH protein pression in 6 T-CEM and J.gamma1 cells after the effects of MBZ. Robust inhibition. **C** Flow cytometry revealed that MBZ treatment induced more apoptotic cells in a dose-dependent manner. **D** Relevant bioluminescence imaging of D12, D26 and D32 days in MBZ group and control group. **E** Histogram shows the bioluminescence signal values for both groups of mice at different time points. **F** Mice of the MBZ group exhibited prolonged survival time compared to the vechile group. **G** Different sizes and weights of liver and spleen, from the MBZ group and vechile group

**Fig. 8:**
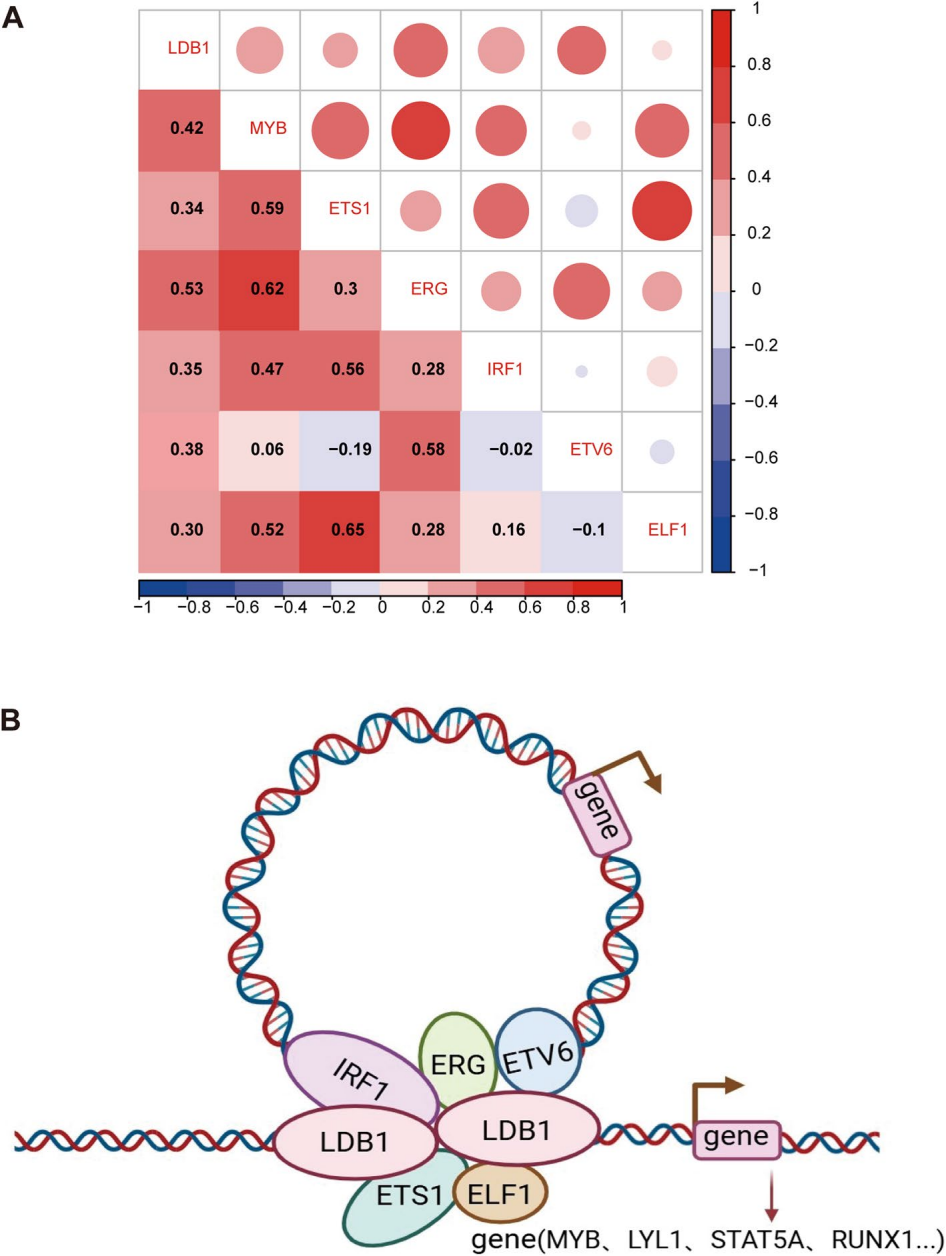
Pattern diagram**. A** An association between LDB1 and master transcription factors through the analysis of transcriptomic data from T-ALL patients in our center. **B.** LDB1 modulates the expression of downstream target gene MYB in T-ALL cells by cooperating with hematopoietic transcription factors -ERG,ETV6 and IRF1

Prior research conducted have reported that MBZ can impede the proliferation of T-ALL cell lines by suppressing the expression of MYB. We initially validated the expression of MYB in five T-ALL cell lines (Jurkat, 6 T-CEM, J.gamma1, CCRF, Molt4) using Western blot analysis (Fig. S11A). Subsequently, we employed MBZ for CCK-8 assays to assess the effects of MBZ. Robust inhibition of tumor cell growth was observed in all four T-ALL cell lines (Fig. 7A). To study the impact of MBZ-mediated MYB degradation on T-ALL cell lines, we measured the protein levels of MYB in T-ALL cell lines treated with increasing concentrations of MBZ. Effective dose-dependent degradation of MYB was observed in both 6 T-CEM and J.gamma1 cell lines. Meanwhile, the expression of the apoptosis protein PARP exhibited a progressive rise according to the dose applied (Fig. 7B and Fig. S11B). Additionally, flow cytometry revealed that MBZ treatment caused a higher number of cells undergoing apoptosis in positively correlation with the dosage. (Fig. 7C and Fig. S11C). Meanwhile, we further treated NC and LDB1 knockdown group cells with 0.5 µM MBZ. The results showed that MBZ induced a higher proportion of apoptosis in the LDB1 knockdown group compared to the NC group (Fig. S12A-B). To further validate the in vivo anticancer activity of MBZ, we established a T-ALL mouse model using 6 T-CEM cells. In comparison to the control group, mice subjected to MBZ treatment demonstrated significantly reduced infiltration in the liver, spleen, and bone marrow (Fig. 7D and Fig. S11D). Tumor bioluminescence histograms demonstrated a much lower signal intensity in the MBZ group than in the control group (Fig. 7E). By conducting a comparison of the survival duration between the two sets of mice, it was demonstrated that the MBZ group had the potential to extend the life expectancy of mice (Fig. 7F). Upon performing dissections on the mice to obtain liver and spleen samples, it was observed that the MBZ-treated group exhibited a notable reduction in the size and weight of their liver and spleen compared to the control group. (Fig. 7G and Fig.S11E). Additionally, the H&E staining analysis of the mouse liver, spleen, and bone marrow demonstrated a notable reduction in tumor cells within the LDB1 knockdown group in comparison to the control group (Fig. S11F). Furthermore, an association between LDB1 and master transcription factors was established through the analysis of transcriptomic data from T-ALL patients in our center (Fig. 8A, Figure.S9A-F, Supplementary Table 13), providing clinical evidence of mutual interactions among transcription factors. Mechanistic insights reveal that LDB1 cooperates with ERG, ETV6, and IRF1 to modulate the expression of downstream effector genes. LDB1 controls MYB through remote enhancer modulation, providing valuable mechanistic insights into its involvement in the progression of T-ALL (Fig. 8B).

## Discussion

T-ALL is driven by oncogenic transcription factors and secondary acquired mutations, collectively contributing to the dysregulation of active signaling pathways. Thus, a current challenge is to gain precise understanding of how these unique genomic elements function in T-ALL pathogenesis, while clarifying mechanisms of gene regulation and identifying new targets for therapeutic intervention of T-ALL [21].

Recent research indicates that LDB1, together with various important transcription factors, forms transcription factor complexes that play crucial roles in cellular fate and function through transcriptional regulation [10]. Initially characterized as a binding partner for LIM domain proteins, LDB1's role as a transcription cofactor is well-established, and its early loss during development leads to embryonic lethality [11]. LDB1 has been confirmed to regulate cardiac myocyte differentiation by activating the MEF2C promoter [16]. Moreover, LDB1 plays an irreplaceable role in early development of the posterior forebrain and thalamus [12]. In mammalian hippocampal cells, the evolutionarily conserved Lhx2-Ldb1 interaction regulates hippocampal cell fate and acquisition of regional identity [22]. Ailen S. Cervino etal. confirmed the conservative and essential role of the Ldb1-Lhx1-Ssbp transcription complex in pre-renal development [23].

Beyond its critical roles in development, LDB1 is implicated in cancer progression. In glioblastoma stem cells, LDB1 is essential for LMO2-driven STAT3 signaling activation. LDB1-LMO2 and the receptor complex composed of gp130-JAK1/2 play a crucial role in signaling cascade activation in glioblastoma stem cells [24]. Previous studies indicate a unique role for LDB1 in neuro-induced breast tumor formation. Nuclear proteins LMO4, LDB1, and SSBP2 and/or SSBP3 participate in cancer cell proliferation, invasion, migration, and angiogenesis, suggesting these nuclear proteins are vital regulators of head and neck squamous cell carcinoma (HNSCC) growth and metastasis [25]. Upregulation of LDB1 expression is correlated with poor prognosis in colorectal cancer [26]. Recently, research on LDB1 in hematologic malignancies has gained attention. In a T-ALL mouse model, LDB1 is essential for Lmo2 oncogene-induced thymocyte self-renewal and T-cell leukemia. Lmo2-induced thymocyte self-renewal is controlled by Ldb1/Lmo2-nucleated transcription complexes which include Hhex, Lyl1, and Nfe2 [20]. A study in MEL cells suggests that the FLI-1/LDB1 complex primarily binds to active enhancers, synergistically activating the expression of megakaryocytic genes and promoting terminal megakaryocyte differentiation [27]. A model study on fetal and adult mice has demonstrated that Ldb1 is essential and consistently involved in the maintenance of hematopoietic stem cells. In addition, Ldb1/Lmo2 complexes positively autoregulate the expression of Lmo2, Lyl1, Tal1, and Gata2 in hematopoietic progenitors [28]. Previous study of jurkat cells revealed that transcription factor of binding in the aberrant MYB-initiated enhancer complex interacts with the promoter region of LMO1, thus driving high levels of oncogene expression [29]. Additionally, In T-cell leukemia, LDB1 directly contributes to the stability of the LMO2 oncoprotein, protecting it from degradation [30]. Similar to the aforementioned transcription factor complexes, our study results suggest that the knockout of the LDB1 gene exerts a profound inhibitory effect on cell proliferation and induces apoptosis in human T-ALL cell lines. The T-ALL cell lines utilized in our experiments include Jurkat, 6 T-CEM, J.gamma1, Molt4, PF-382, and others. Significant mutations and drivers in these cell lines are available online at: https://humantallcelllines.wordpress.com/comprehensivetable/. In a mouse model, the suppression of LDB1 expression profoundly inhibits tumor growth and enhances overall survival. Additionally, our analysis of transcriptomic data from T-ALL patients at our center reveals a correlation between the expression levels of core hematopoietic transcription factors (ERG, ETV6, ETS1, RUNX1, IRF1, and MYB) and LDB1, providing further clinical validation of potential interactions among transcription factor complexes. We propose that LDB1, in coordination with core transcription factors, forms transcription factor complexes to jointly regulate the expression of genes related to hematopoietic stem cell differentiation, thereby promoting the occurrence and development of T-ALL.

MYB exerts a pivotal role in regulating the proliferation and differentiation of hematopoietic stem cells and serves as a central component of complexes responsible for maintaining abnormal gene expression in various leukemias, including AML, CML, and ALL[31, 32]. Aberrant expression of the oncogene MYB in infant and adult T-cell leukemia/lymphoma cells leads to dysregulation of the c-MYB signaling pathway, laying the foundation for the malignancy of tumor cells [33–37]. Almeida etal. confirm that MYB overexpression is a dependency factor and therapeutic target in T-cell leukemia [38]. Despite the potential therapeutic strategy of MYB inhibition emerging for various leukemias, including T-ALL, the exact mechanisms of its transcriptional regulation remain unclear. Moreover, the identification and targeting of enhancers provide additional treatment approaches. Previous research has reported that the LDB1 complex dynamically binds to a distal enhancer in the Myb-Hbs1l intergenic region upstream of the MYB gene, initiating MYB through chromatin looping as a transcription elongation factor [39]. Similar report have confirmed that the LDB1 complex indirectly regulates the interaction between MYB enhancers and the LDB1 complex by controlling distant enhancers [40]. Interestingly, we discovered a proximal enhancer fragment near the downstream region of the MYB gene, controlled by the AHI1 enhancer, which regulates its expression. Silencing this fragment resulted in decreased MYB expression and reduced T-ALL cell proliferation. Previous studies have indicated that MBZ can inhibit the expression of Notch 1 signaling protein in T-ALL cells, suggesting that the Notch 1 pathway may be a significant target of MBZ in T-ALL cells [41]. A recent surprising discovery in T-ALL cells showed that targeting MYB-dependent oncogenic 5' super-enhancers with mithramycin led to MYB protein degradation and T-ALL cell apoptosis [42]. Consistent with this study, our experiments with the MYB inhibitor MBZ in in vitro and in vivo settings demonstrated the anti-tumor effects of MBZ, providing new insights and perspectives for the diagnosis and treatment of clinical T-ALL patients.

## Conclusions

In summary, our research results identify LDB1 as a key player in T-ALL progression. Mechanistic studies suggest that LDB1, in coordination with ERG, ETV6, ETS1, RUNX1, IRF1, and MYB, regulates the expression of downstream target genes. Furthermore, LDB1 regulates MYB through remote control of enhancer sequences, providing mechanistic insights into its role in T-ALL progression.

Novelty and Limitation

### Novelty and Limitation

Our study has revealed a novel mechanism through which LDB1 drives the progression of T-cell acute lymphoblastic leukemia (T-ALL). We have demonstrated that LDB1 collaborates with ERG, ETV6, and IRF1 to regulate enhancer activity and control the expression of MYB. Furthermore, our findings underscore the therapeutic potential of MBZ, a novel inhibitor of MYB, providing a new strategy for combating T-ALL. Unfortunately, we primarily conducted experiments using T-ALL cell lines and have not yet undertaken studies involving transgenic mice, PDX models, or spontaneous model experiments.

## Supplementary Information

Supplementary Material 1. Supplementary Figure 1. A. The white slice indicates that LDB1 knockdown remarkably suppressed the proliferation of Jurkat, 6T-CEM, and J.gamma1 cells compared to scramble cells. B.The proportion of cell proliferation was conducted by EdU-594 assays in the LDB1 knockdown group compared to the control group in 6T-CEM and Jurkat cells five days after virus transfection with the quantitative data depicted in bar graphs

Supplementary Material 2. Supplementary Figure 2. A-B: Knockdown of LDB1 induces cell cycle arrest in the G0/G1 phase in Jurkat and 6T-CEM cells with the quantitative data depicted in bar graphs. C-D: Knockdown of LDB1 increased the apoptotic rates of PF-382 and SUP1 cell lines with the quantitative data depicted in bar graphs

Supplementary Material 3. Supplementary Figure 3. A. H&E staining analysis of the mouse spleen, demonstrated a notable reduction in tumor cells in the LDB1 knockdown group in comparison to the control group. B-D. The proportion of 6T-CEM cells in the liver, bone marrow (BM), and spleen of both control and LDB1 knockdown groups was assessed using hCD45 flow cytometry, with the quantitative data depicted in bar graphs

Supplementary Material 4. Supplementary Figure 4. A. LDB1 gene knockdown validation by PCR verification of 6T-CEM and J.gamma1 cells submitted for sequencing. B. LDB1 protein knockdown validation by Western Blot verification of 6T-CEM and J.gamma1 cells submitted for sequencing. C. The qPCR analysis with cells of six days post-viral transduction showed that the expression levels of genes sustaining the hematopoietic stem cell differentiation, such as NOTCH1, MYB, and RUNX1, were decreased

Supplementary Material 5. Supplementary Figure5. A. a CFU assay on Jurkat and 6T-CEM cell lines showing dysregulated self-renewal  in LDB1-knockdown T-ALL cell. B. stem cell marker analysis on Jurkat cell beads using flow cytometry

Supplementary Material 6. Supplementary Figure6. Pie chart exhibiting the DNA binding sites distribution for different antibodies in 6T-CEM cells using CUT&Tag experiments. A. ERG antibody/6T-CEM cells. B. IRF1 antibody/6T-CEM cells. C. ETV6 antibody/6T-CEM cells

Supplementary Material 7. Supplementary Figure7. Pie chart exhibiting the DNA binding sites distribution for H3K27AC antibody in sh-NC and sh-LDB1 of 6T-CEM cells using CUT&Tag experiments. A. H3K27AC antibody/sh-NC and sh-LDB1 of 6T-CEM cells .B. After knocking down the expression level of LDB1, the enrichment of H3K27ac at the positions where LDB1 binds on the chromatin is reduced

Supplementary Material 8. Supplementary Figure 8. The correlation between LDB1,ERG,ETV6 and IRF1. A. An IGV plot of CUT&Tag data illustrates the co-occupancy  of LDB1,ERG,ETV6,and IRF1 at the SEs regions of ERG. B. An IGV plot of CUT&Tag data illustrates the co-occupancy  of LDB1,ERG,ETV6,and IRF1 at the SEs regions of ETV6. C. An IGV plot of CUT&Tag data illustrates the co-occupancy  of LDB1,ERG,ETV6,and IRF1 at the SEs regions of IRF1. D. An IGV plot of CUT&Tag data illustrates the co-occupancy  of LDB1,ERG,ETV6,and IRF1 at the SEs regions of LDB1

Supplementary Material 9. Supplementary Figure 9. Correlation analysis of the expression levels of LDB1 with ERG,ETV6,ETS1,MYB and IRF1 based on pediatric T-ALL patients

>Supplementary Material 10. Supplementary Figure 10. A.IGV visual analysis highlights that the binding profile of LDB1 complex in Loucy, Molt-4, and J.gamma1 cell lines  similarly reveals that there is binding at distal enhancers in the Myb-AHI1 intergenic region. B. Genomic features (%) of LDB1 peaks in in Loucy, Molt-4, and J.gamma1 cell lines.

Supplementary Material 11. Supplementary Figure  11. A. Western blotting analysis showed that MYB protein pression in T-ALL cells. B. Western blotting analysis showed that MYB, PARP and GAPDH protein pression in Jurkat and CCRF cells after the effects of MBZ. Robust inhibition. C. Flow cytometry revealed that MBZ treatment induced more apoptotic cells in a dose-dependent manner in Jurkat and CCRF cells. D. Histogram shows the bioluminescence signal values for both groups of mice at liver, spleen and bone marrow. E. Different sizes and weights of liver and spleen, from the MBZ group and control group. F. Representative images of HE staining of mice livers, spleens and bone marrows

Supplementary Material 12. Supplementary Figure 12. A-B: Flow cytometry revealed that MBZ induced a higher proportion of apoptosis in the LDB1 knockdown group compared to the NC group in 6T-CEM and J.gamma1 cells

Supplementary Material 13. Supplementary Table 1. The sequences of sh-LDB1

Supplementary Material 14. Supplementary Table 2. Details of all PCR primers utilized in this study

Supplementary Material 15. Supplementary Table 3. Information on all antibodies utilized in this study

Supplementary Material 16. Supplementary Table 4. CRISPR screen data indicated the dependence of T-ALL cell line Jurkat on LDB1

Supplementary Material 17. Supplementary Table 5. Differentially expressed genes detected by RNA-Seq in 6T-CEM cells following LDB1 knockout

Supplementary Material 18. Supplementary Table 6. Differentially expressed genes detected by RNA-Seq in J.gamma1 cells following LDB1 knockout

Supplementary Material 19. Supplementary Table 7. GSEA analysis results following LDB1 knockout in 6T-CEM cells

Supplementary Material 20. Supplementary Table 8. GSEA analysis results following LDB1 knockout in J.gamma1 cells

Supplementary Material 21. Supplementary Table 9. The peaks identified by CUT&Tag analysis of LDB1 in 6T-CEM cells

Supplementary Material 22. Supplementary Table 10. The peaks identified by CUT&Tag analysis of ERG in 6T-CEM cells

Supplementary Material 23. Supplementary Table 11. The peaks identified by CUT&Tag analysis of IRF1 in 6T-CEM cells

Supplementary Material 24. Supplementary Table 12. The peaks identified by CUT&Tag analysis of ETV6 in 6T-CEM cells

Supplementary Material 25. Supplementary Table 13. Transcriptomic sequencing expression levels of various transcription factors in T-ALL patients

Supplementary Material 26. Supplementary Table 14. The sequences of sg-NC and sgRNA

Supplementary Material 27. Supplementary Table 15. The peaks identified by CUT&Tag analysis of LDB1 in J.gamma1cells

Supplementary Material 28. Supplementary Table 16. The peaks identified by CUT&Tag analysis of LDB1 in Loucy cells

Supplementary Material 29. Supplementary Table 17. The peaks identified by CUT&analysis of LDB1 in Molt-4 cells

## Data Availability

The data that support the findings of this study are available on reasonable request from the corresponding author. RNA‑seq and CUT-TAG data have been submitted to the GEO database with Accession Number GSE (GSE253038 and GSE252992).

## Abbreviations

T-ALL: T-cell acute lymphoblastic leukemia
OS: Overall survival
LDB1: LIM domain-binding protein 1
FBS: Fetal bovine serum
GSEA: Gene Set Enrichment Analysis
CCLE: Cancer Cell Line Encyclopedia
CUT&Tag: Cleavage Under Targets and Tagmentation
sgRNAs: Single guide RNAs
MBZ: Mebendazole
