# Supplementary figures and images for: Enhancer looping protein LDB1 modulates MYB expression in T-ALL cell lines in vitro by cooperating with master transcription factors
Source: J Exp Clin Cancer Res. 2024 Oct 9;43:283. doi: 10.1186/s13046-024-03199-1 (PMC11462673; doi:10.1186/s13046-024-03199-1)

**A**

sh-NC

sh-LDB1#1

sh-LDB1#2

Jurkat

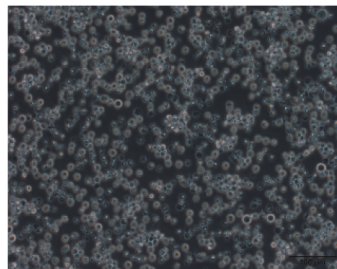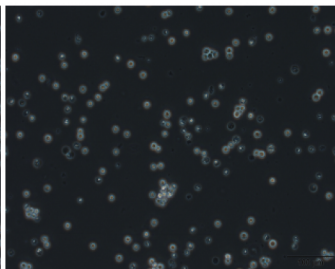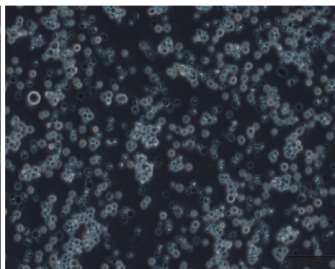

6T-CEM

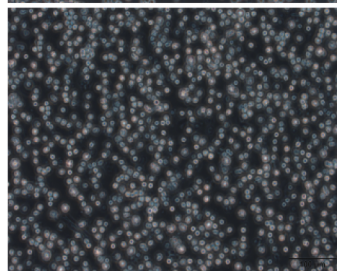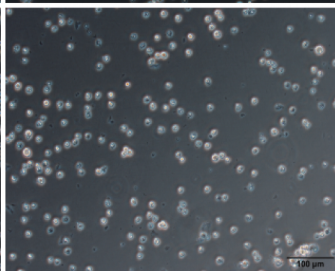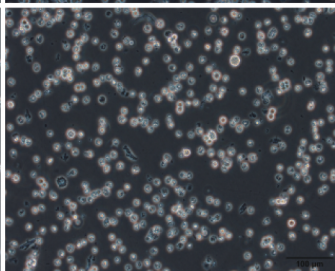

J.gamma1

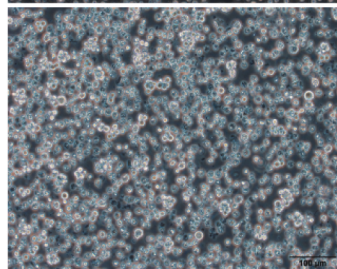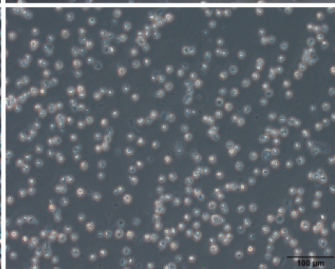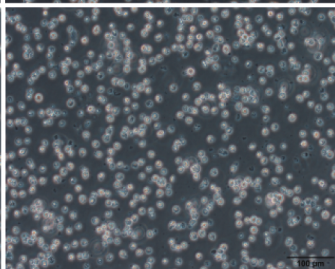**B**

Jurkat

sh-NC

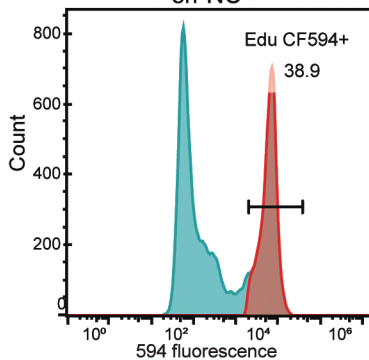

sh-LDB1

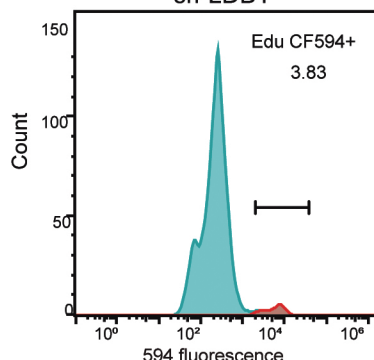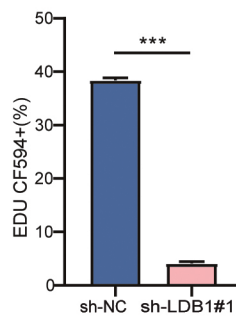

6T-CEM

sh-NC

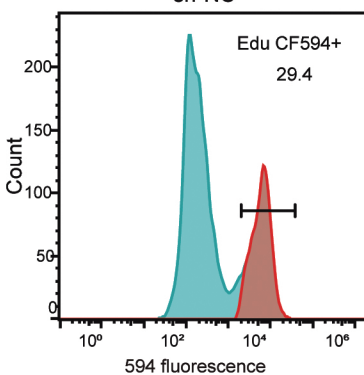

sh-LDB1

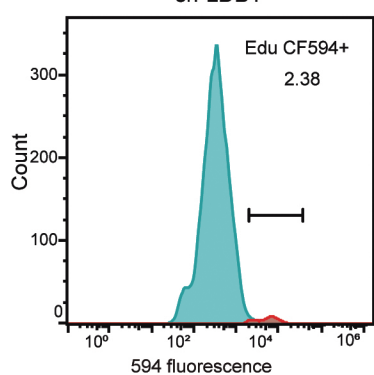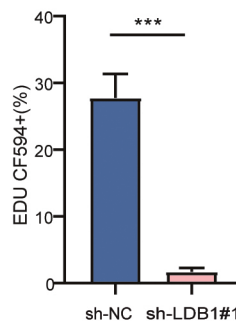

Supplement: Supplementary file 1 — Supplementary Material 1. Supplementary Figure 1. A. The white slice indicates that LDB1 knockdown remarkably suppressed the proliferation of Jurkat, 6T-CEM, and J.gamma1 cells compared to scramble cells. B.The proportion of cell proliferation was conducted by EdU-594 assays in the LDB1 knockdown group compared to the control group in 6T-CEM and Jurkat cells five days after virus transfection with the quantitative data depicted in bar graphs [file 13046_2024_3199_MOESM1_ESM.pdf]

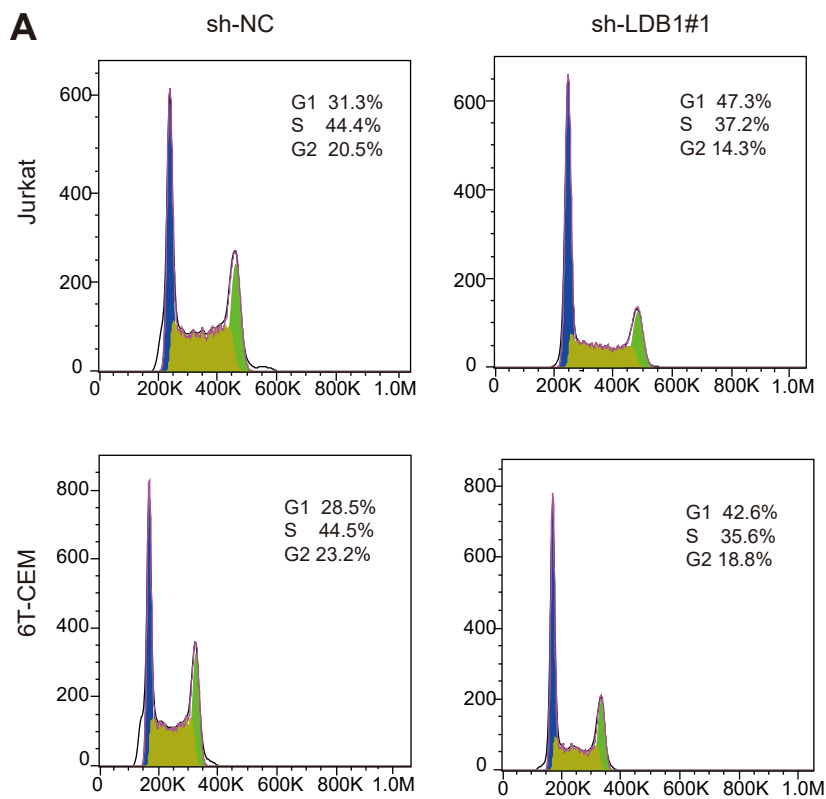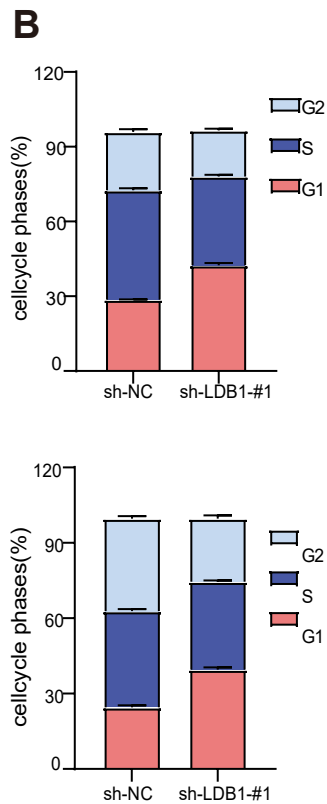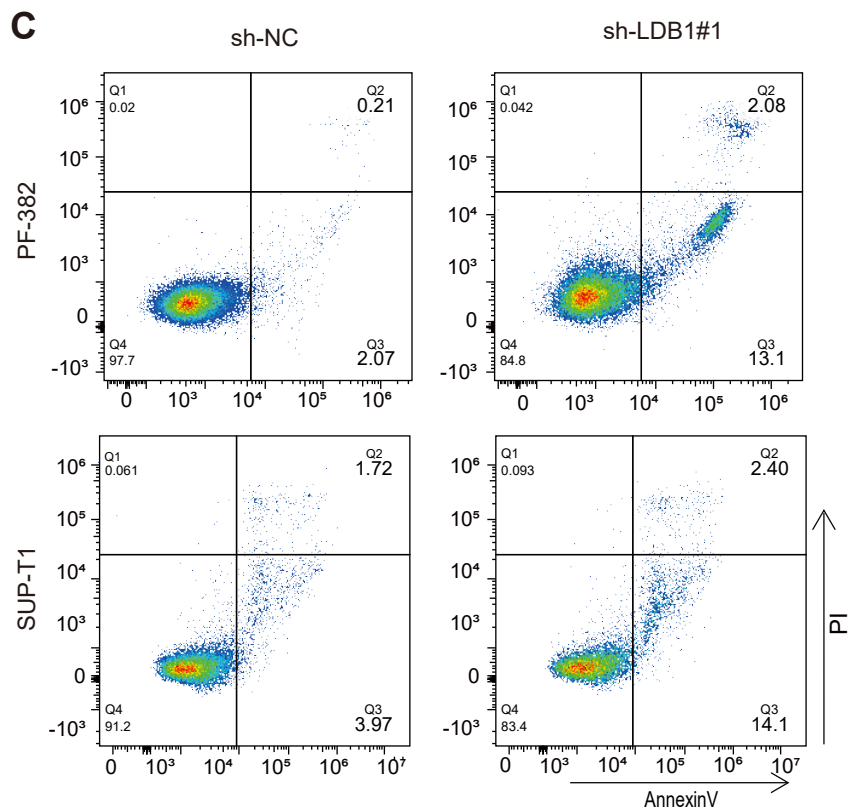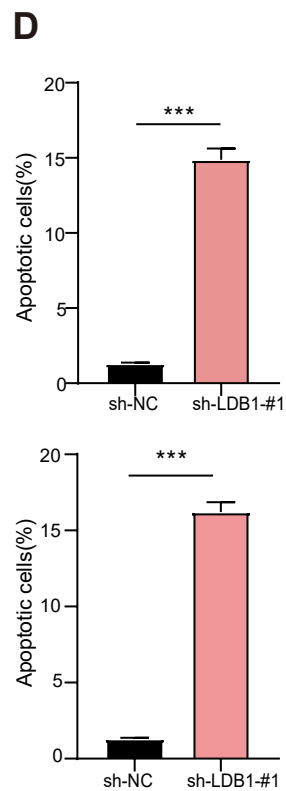

Supplement: Supplementary file 2 — Supplementary Material 2. Supplementary Figure 2. A-B: Knockdown of LDB1 induces cell cycle arrest in the G0/G1 phase in Jurkat and 6T-CEM cells with the quantitative data depicted in bar graphs. C-D: Knockdown of LDB1 increased the apoptotic rates of PF-382 and SUP1 cell lines with the quantitative data depicted in bar graphs [file 13046_2024_3199_MOESM2_ESM.pdf]

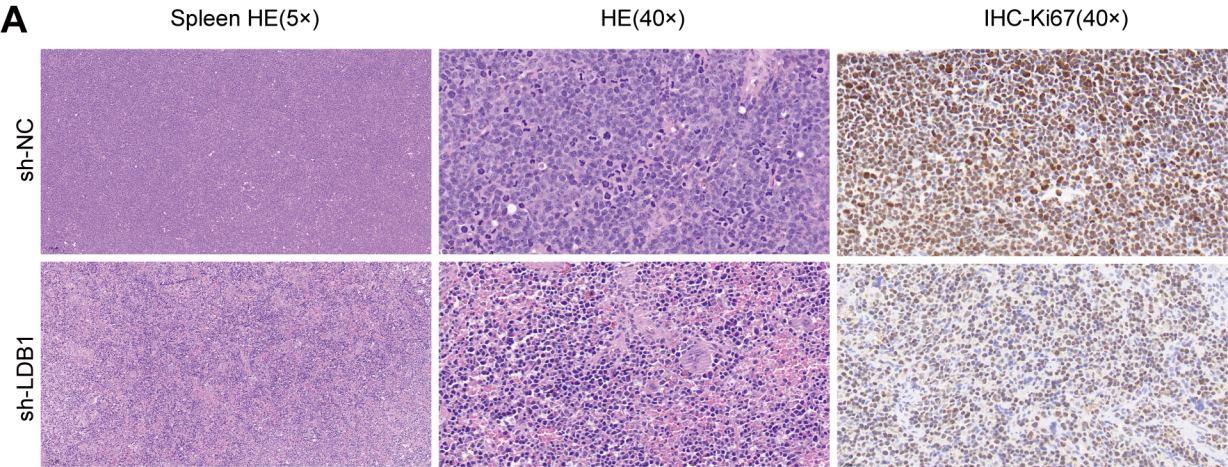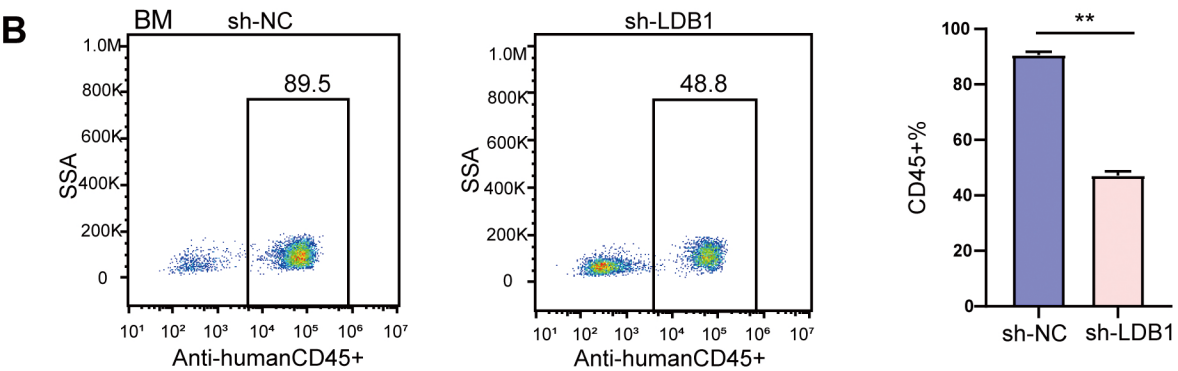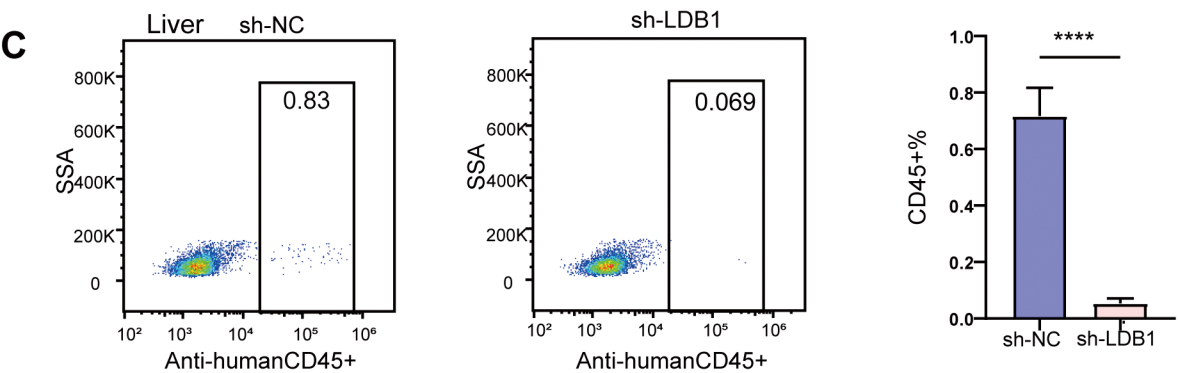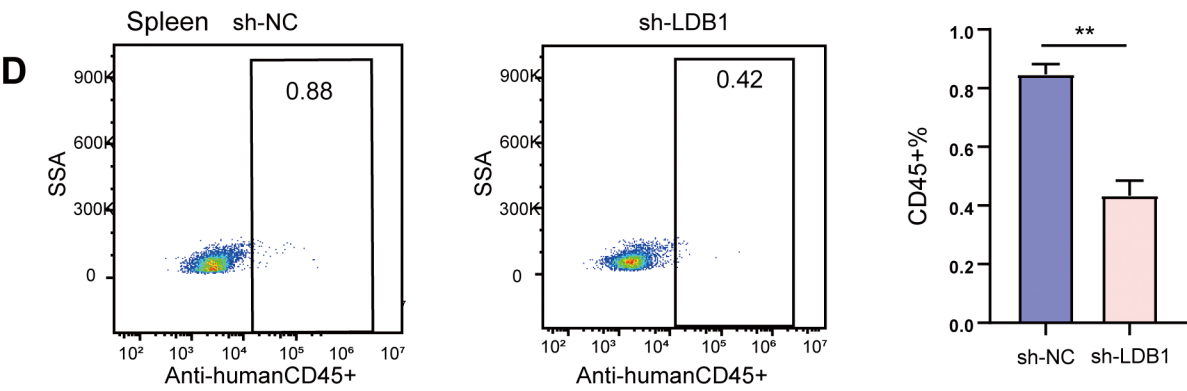

Supplement: Supplementary file 3 — Supplementary Material 3. Supplementary Figure 3. A. H&E staining analysis of the mouse spleen, demonstrated a notable reduction in tumor cells in the LDB1 knockdown group in comparison to the control group. B-D. The proportion of 6T-CEM cells in the liver, bone marrow (BM), and spleen of both control and LDB1 knockdown groups was assessed using hCD45 flow cytometry, with the quantitative data depicted in bar graphs [file 13046_2024_3199_MOESM3_ESM.pdf]

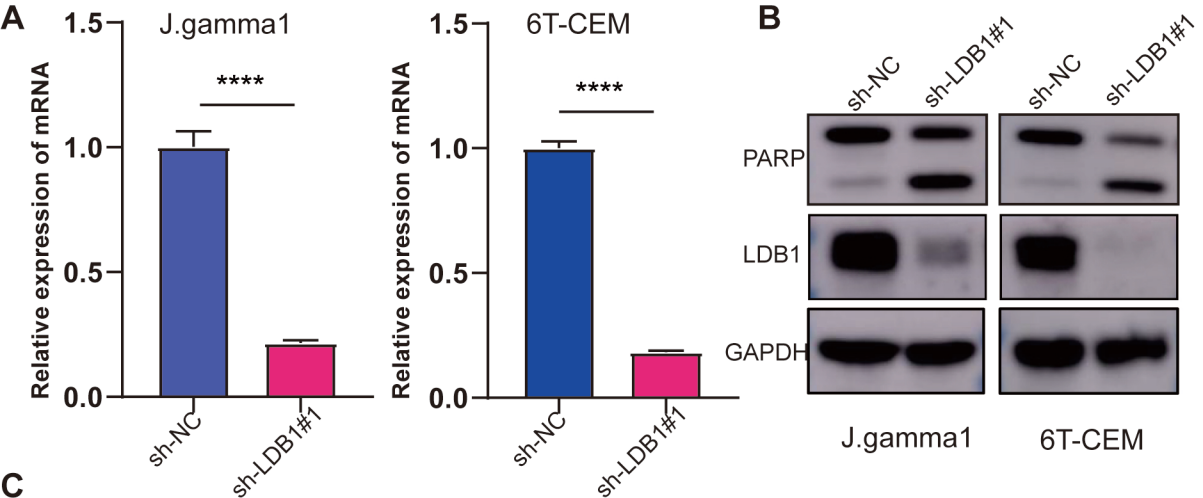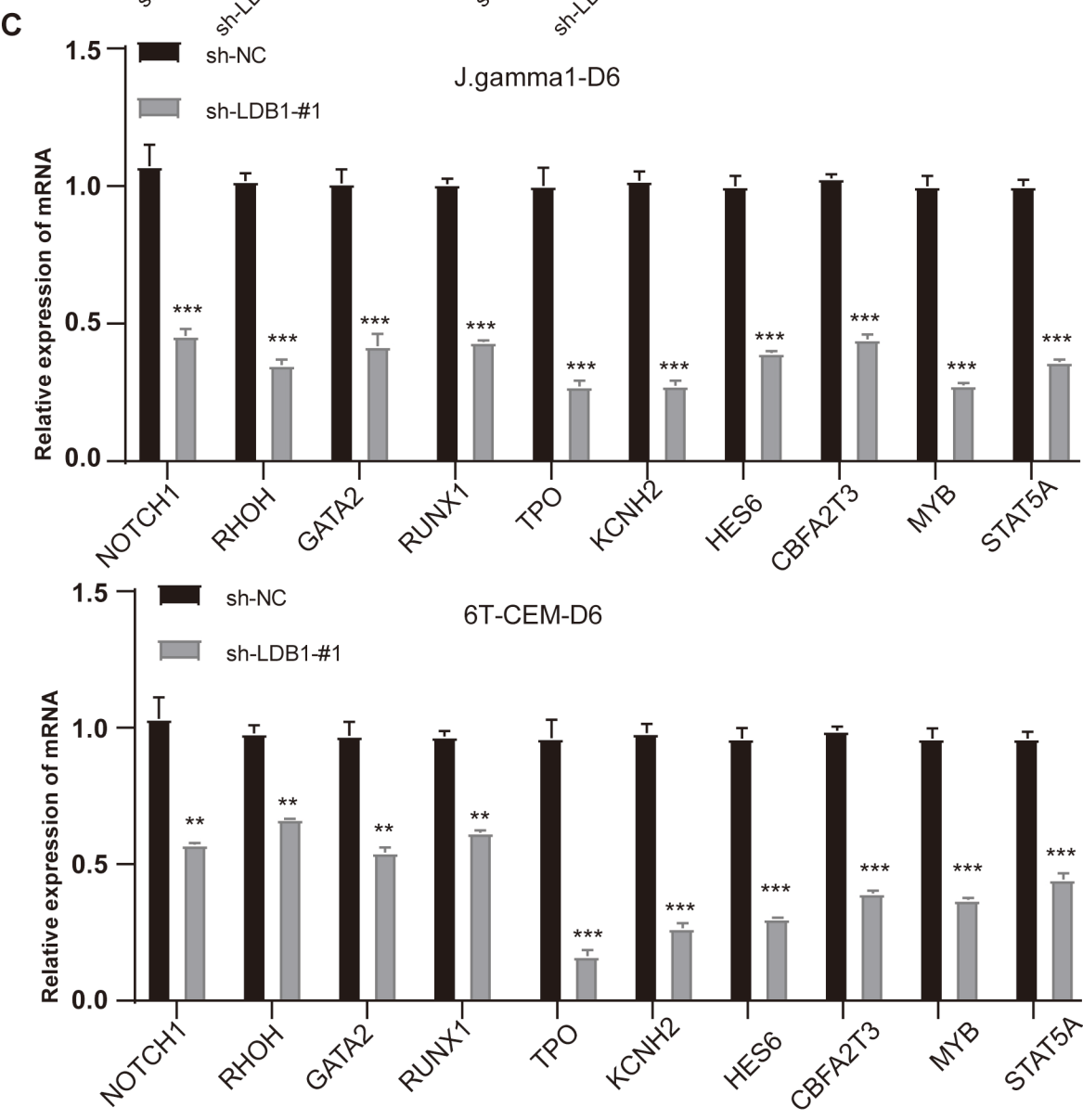

Supplement: Supplementary file 4 — Supplementary Material 4. Supplementary Figure 4. A. LDB1 gene knockdown validation by PCR verification of 6T-CEM and J.gamma1 cells submitted for sequencing. B. LDB1 protein knockdown validation by Western Blot verification of 6T-CEM and J.gamma1 cells submitted for sequencing. C. The qPCR analysis with cells of six days post-viral transduction showed that the expression levels of genes sustaining the hematopoietic stem cell differentiation, such as NOTCH1, MYB, and RUNX1, were decreased [file 13046_2024_3199_MOESM4_ESM.pdf]

**A**

sh-NC

sh-LDB1

Jurkat

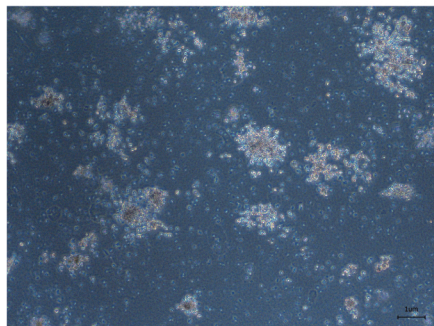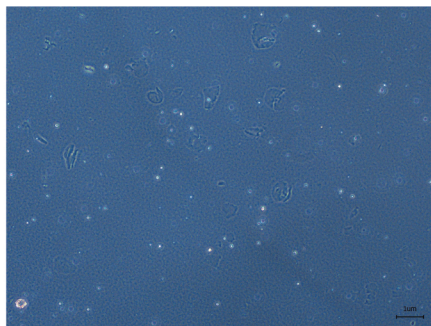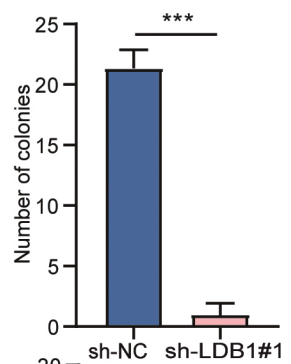

PF-382

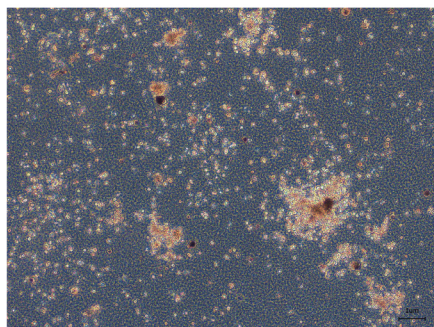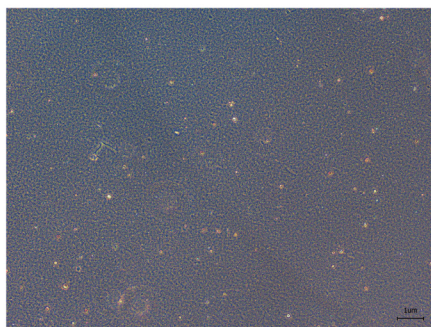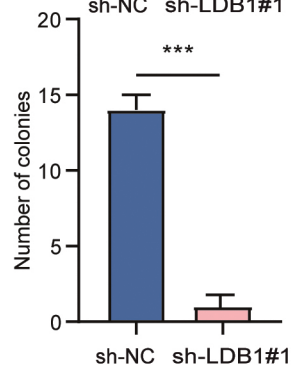**B**

sh-NC

sh-LDB1

Jurkat

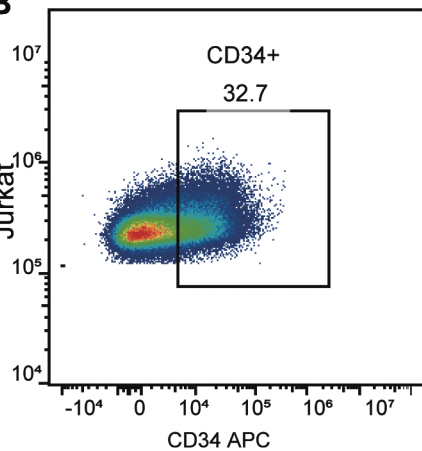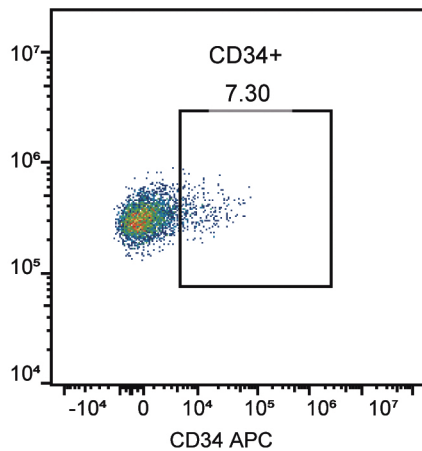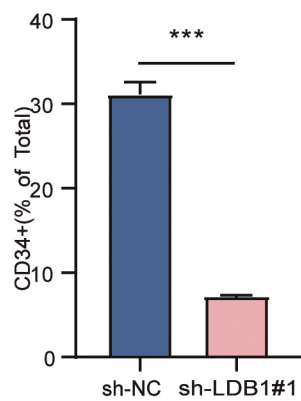

PF-382

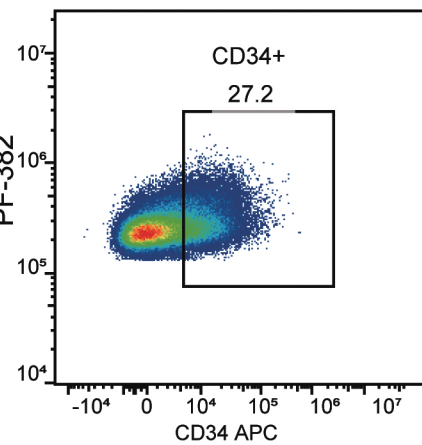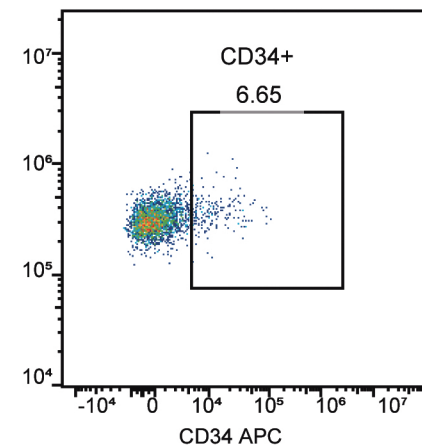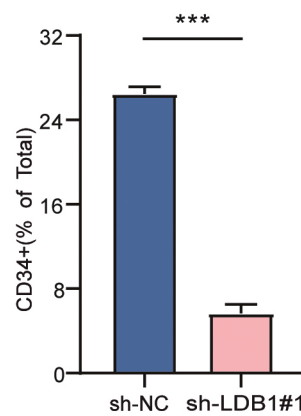

Supplement: Supplementary file 5 — Supplementary Material 5. Supplementary Figure5. A. a CFU assay on Jurkat and 6T-CEM cell lines showing dysregulated self-renewal in LDB1-knockdown T-ALL cell. B. stem cell marker analysis on Jurkat cell beads using flow cytometry [file 13046_2024_3199_MOESM5_ESM.pdf]

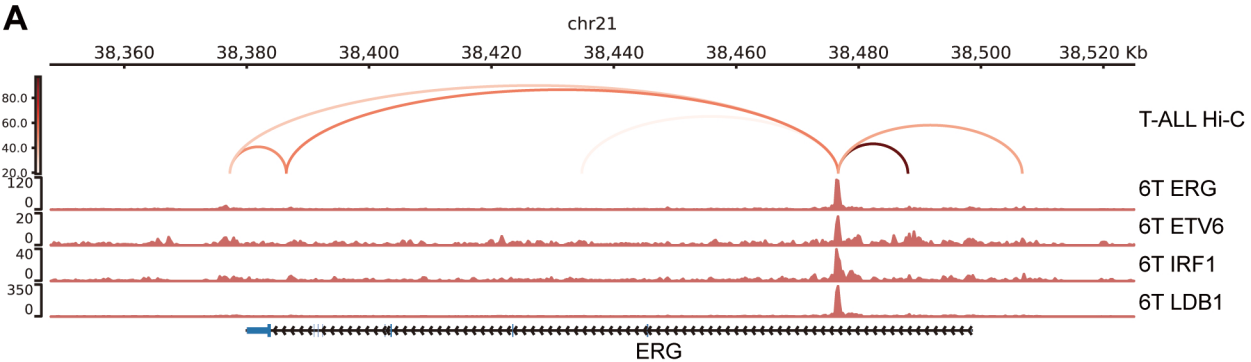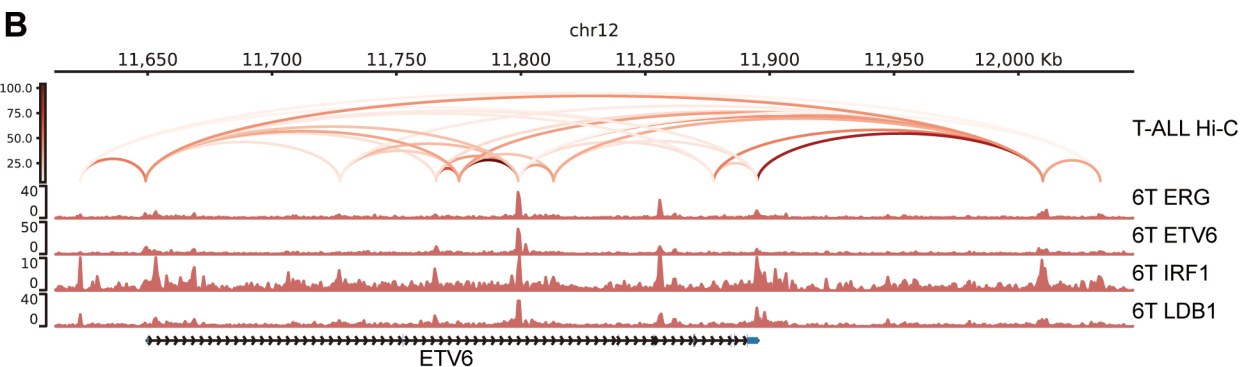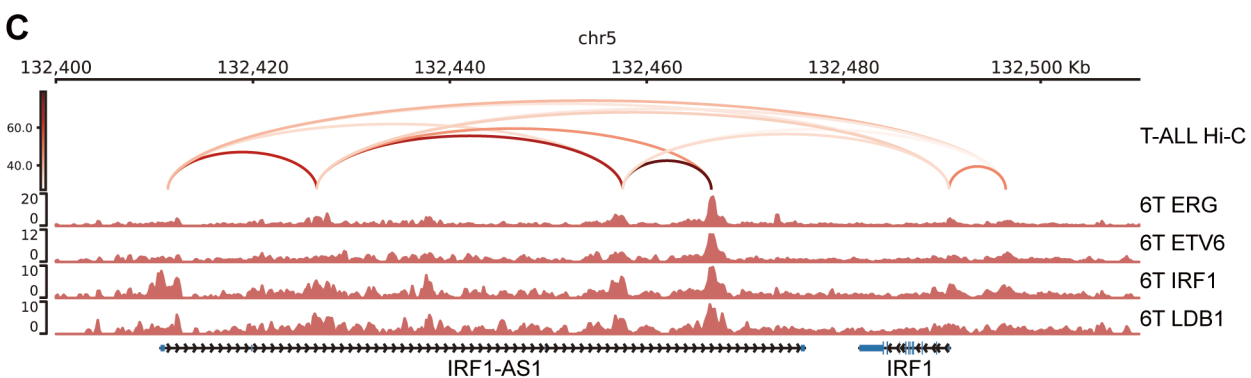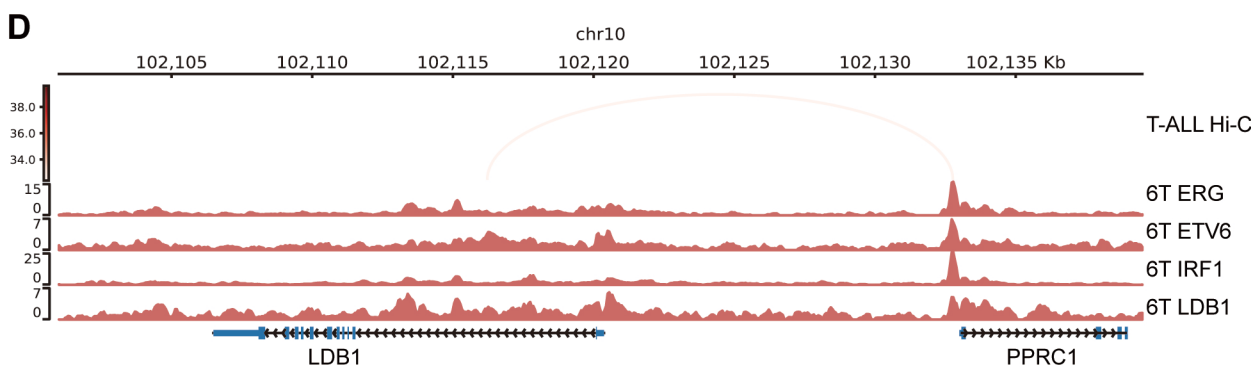

Supplement: Supplementary file 8 — Supplementary Material 8. Supplementary Figure 8. The correlation between LDB1,ERG,ETV6 and IRF1. A. An IGV plot of CUT&Tag data illustrates the co-occupancy of LDB1,ERG,ETV6,and IRF1 at the SEs regions of ERG. B. An IGV plot of CUT&Tag data illustrates the co-occupancy of LDB1,ERG,ETV6,and IRF1 at the SEs regions of ETV6. C. An IGV plot of CUT&Tag data illustrates the co-occupancy of LDB1,ERG,ETV6,and IRF1 at the SEs regions of IRF1. D. An IGV plot of CUT&Tag data illustrates the co-occupancy of LDB1,ERG,ETV6,and IRF1 at the SEs regions of LDB1 [file 13046_2024_3199_MOESM8_ESM.pdf]

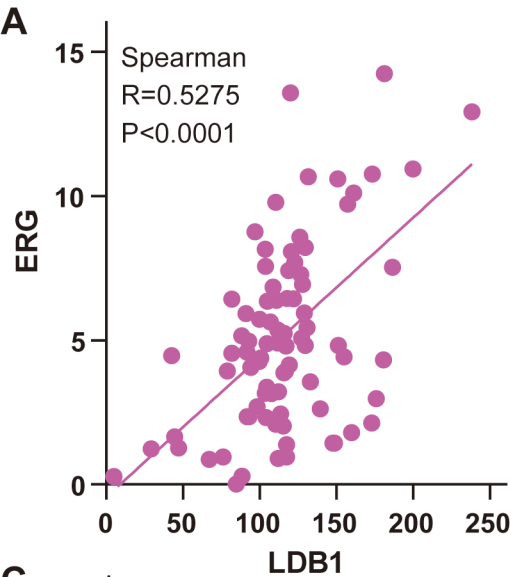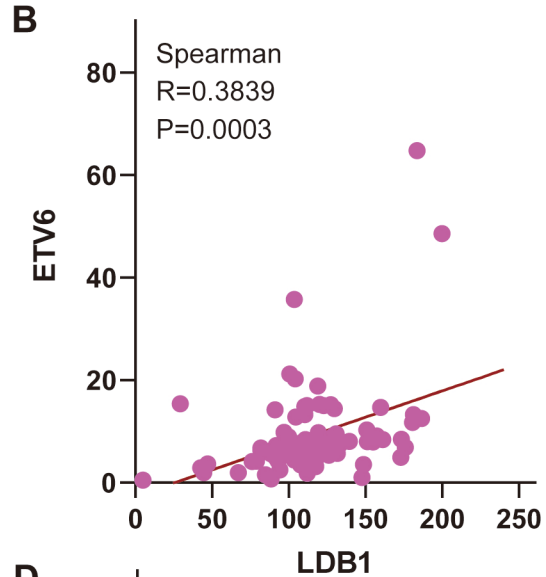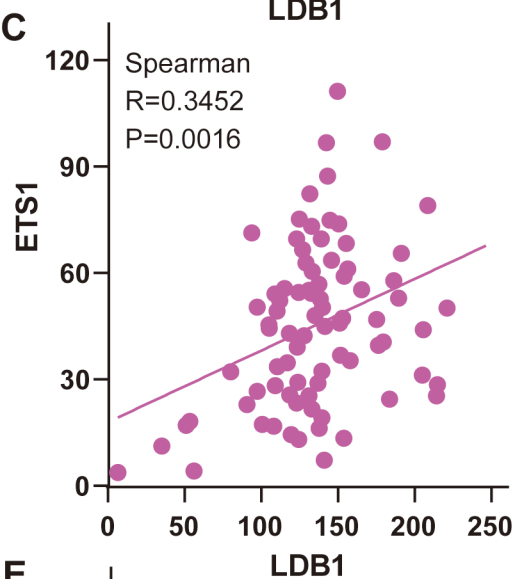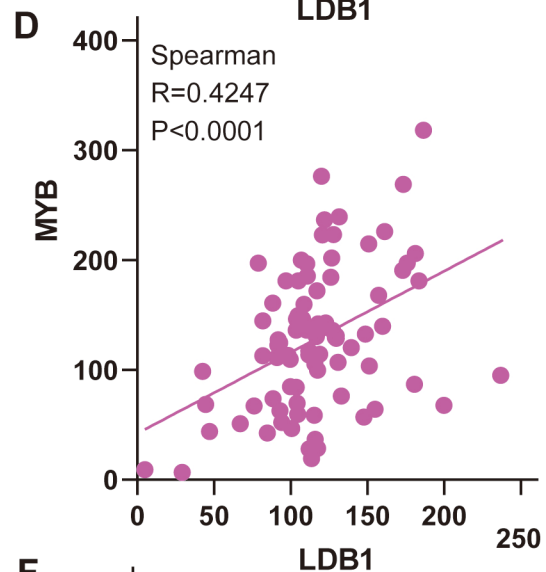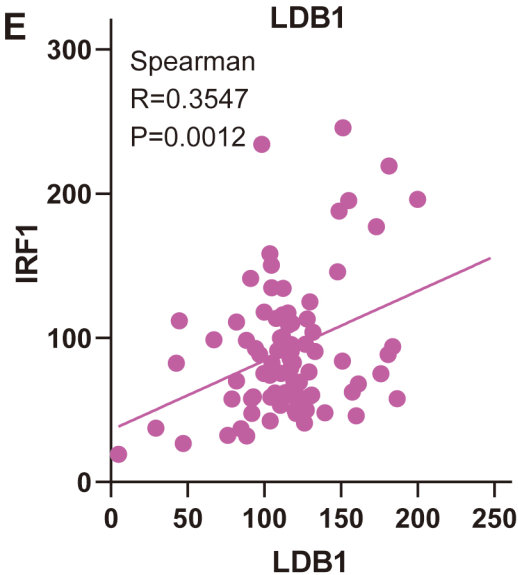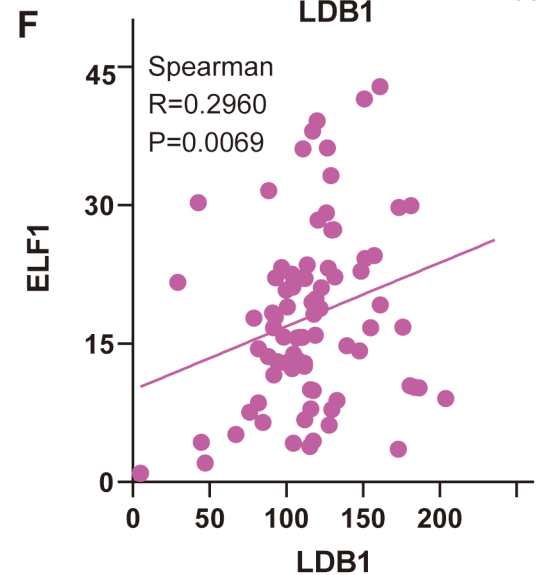

Supplement: Supplementary file 9 — Supplementary Material 9. Supplementary Figure 9. Correlation analysis of the expression levels of LDB1 with ERG,ETV6,ETS1,MYB and IRF1 based on pediatric T-ALL patients [file 13046_2024_3199_MOESM9_ESM.pdf]

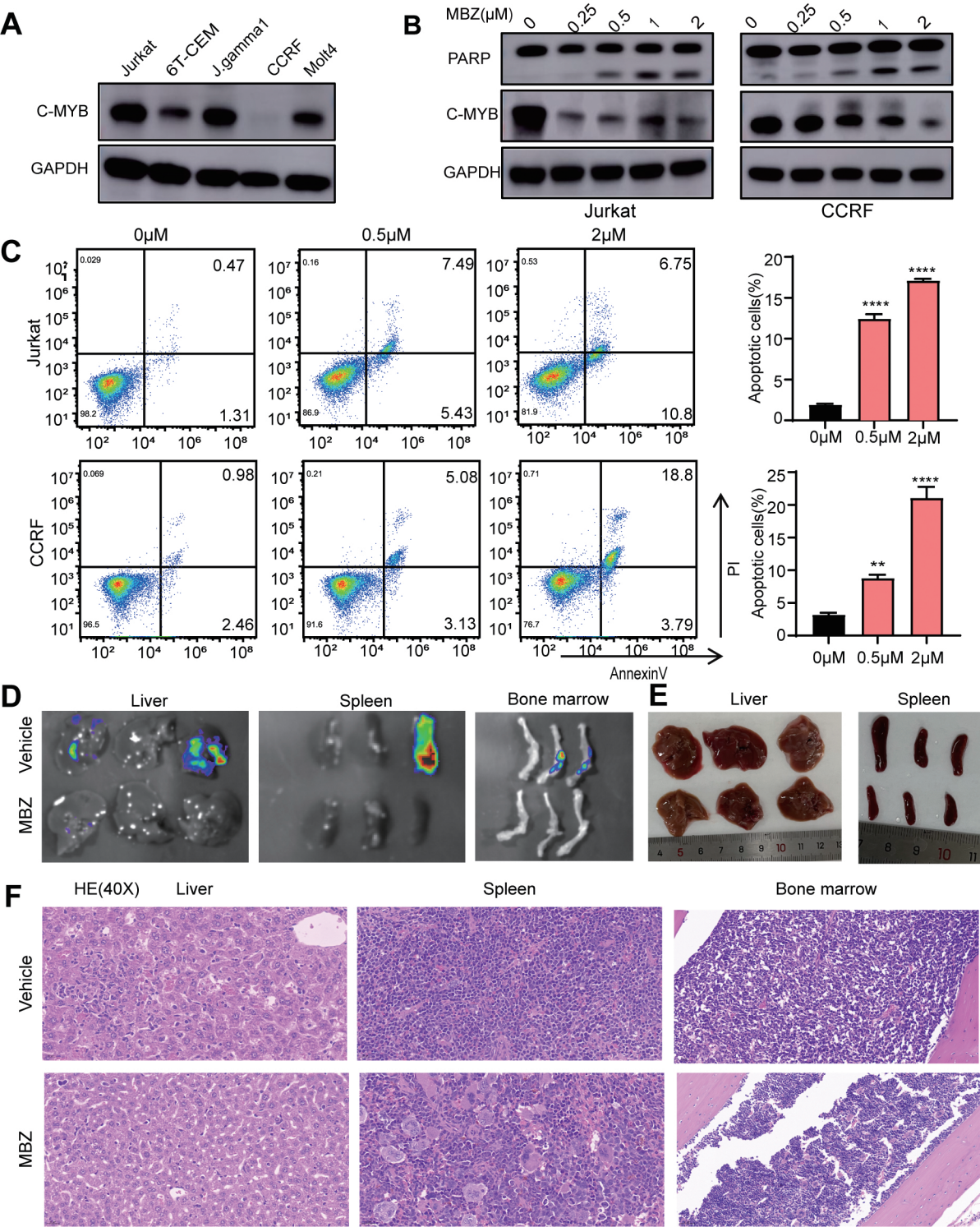

Supplement: Supplementary file 10 — Supplementary Material 11. Supplementary Figure 11. A. Western blotting analysis showed that MYB protein pression in T-ALL cells. B. Western blotting analysis showed that MYB, PARP and GAPDH protein pression in Jurkat and CCRF cells after the effects of MBZ. Robust inhibition. C. Flow cytometry revealed that MBZ treatment induced more apoptotic cells in a dose-dependent manner in Jurkat and CCRF cells. D. Histogram shows the bioluminescence signal values for both groups of mice at liver, spleen and bone marrow. E. Different sizes and weights of liver and spleen, from the MBZ group and control group. F. Representative images of HE staining of mice livers, spleens and bone marrows [file 13046_2024_3199_MOESM10_ESM.pdf]

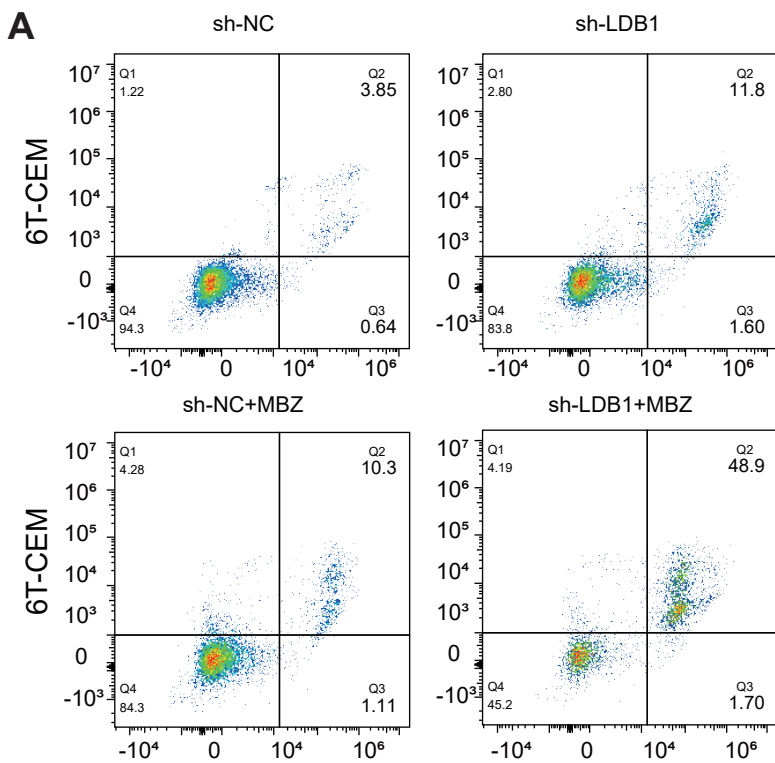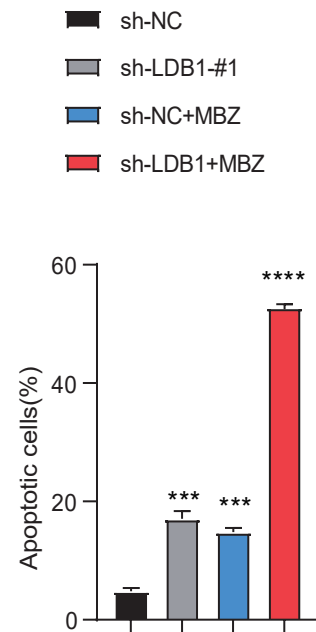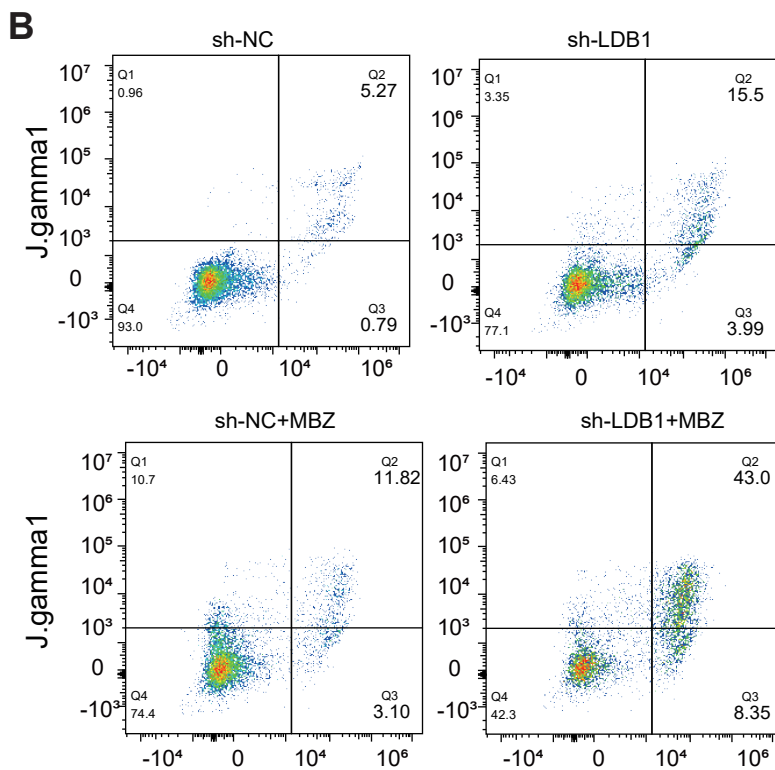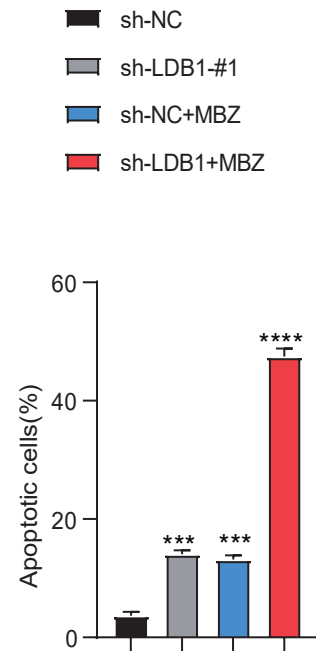

Supplement: Supplementary file 11 — Supplementary Material 12. Supplementary Figure 12. A-B: Flow cytometry revealed that MBZ induced a higher proportion of apoptosis in the LDB1 knockdown group compared to the NC group in 6T-CEM and J.gamma1 cells [file 13046_2024_3199_MOESM11_ESM.pdf]
