# Supplementary material for: Enhancer looping protein LDB1 modulates MYB expression in T-ALL cell lines in vitro by cooperating with master transcription factors
Source: J Exp Clin Cancer Res. 2024 Oct 9;43:283. doi: 10.1186/s13046-024-03199-1 (PMC11462673; doi:10.1186/s13046-024-03199-1)
Supplement: Supplementary file 6 — Supplementary Material 6. Supplementary Figure6. Pie chart exhibiting the DNA binding sites distribution for different antibodies in 6T-CEM cells using CUT&Tag experiments. A. ERG antibody/6T-CEM cells. B. IRF1 antibody/6T-CEM cells. C. ETV6 antibody/6T-CEM cells [file 13046_2024_3199_MOESM6_ESM.pdf]

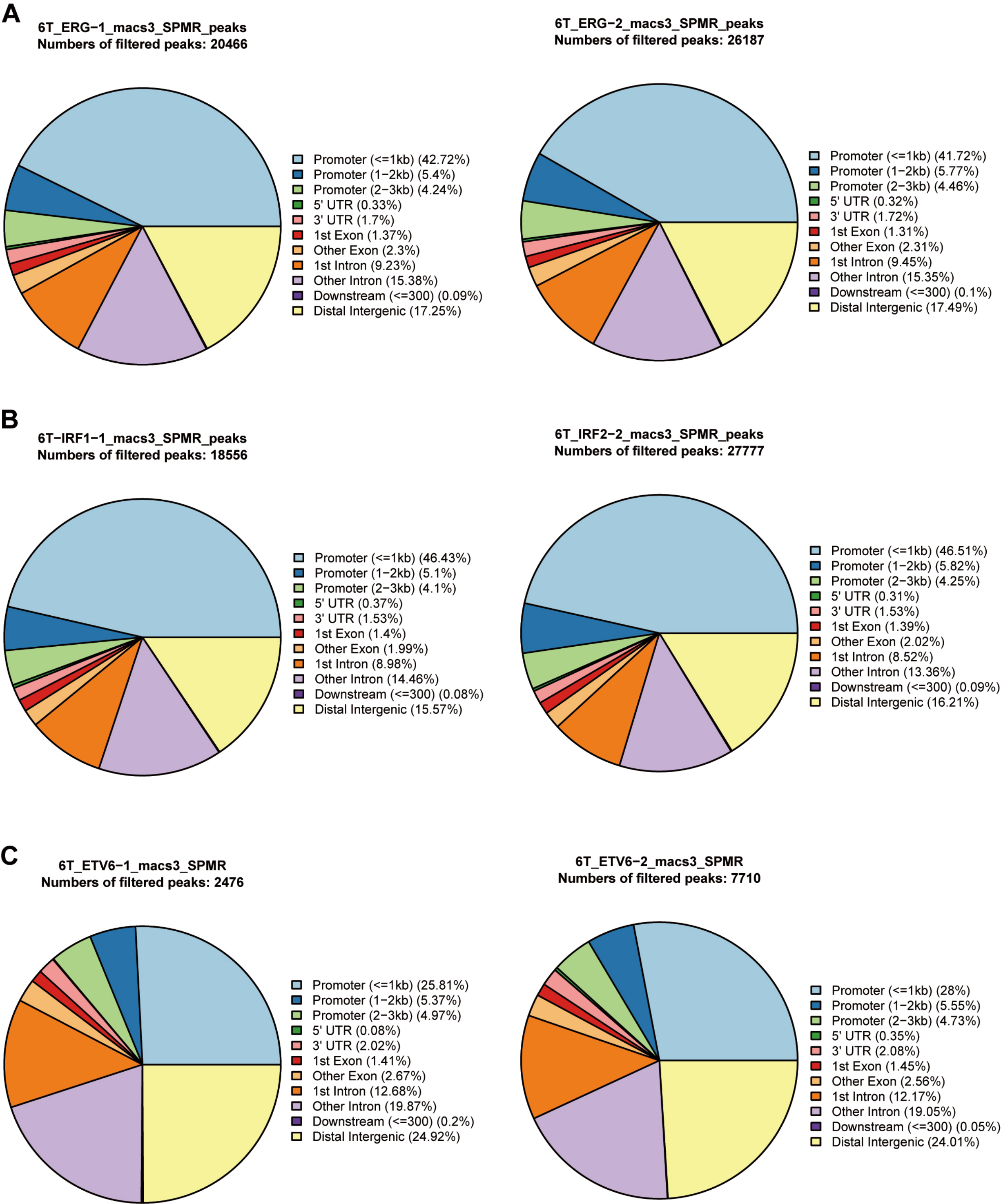
A
6T_ERG−1_macs3_SPMR_peaks
Numbers of filtered peaks: 20466
Promoter (<=1kb) (42.72%)
Promoter (1−2kb) (5.4%)
Promoter (2−3kb) (4.24%)
5' UTR (0.33%)
3' UTR (1.7%)
1st Exon (1.37%)
Other Exon (2.3%)
1st Intron (9.23%)
Other Intron (15.38%)
Downstream (<=300) (0.09%)
Distal Intergenic (17.25%)
6T_ERG−2_macs3_SPMR_peaks
Numbers of filtered peaks: 26187
Promoter (<=1kb) (41.72%)
Promoter (1−2kb) (5.77%)
Promoter (2−3kb) (4.46%)
5' UTR (0.32%)
3' UTR (1.72%)
1st Exon (1.31%)
Other Exon (2.31%)
1st Intron (9.45%)
Other Intron (15.35%)
Downstream (<=300) (0.1%)
Distal Intergenic (17.49%)
B
6T−IRF1−1_macs3_SPMR_peaks
Numbers of filtered peaks: 18556
Promoter (<=1kb) (46.43%)
Promoter (1−2kb) (5.1%)
Promoter (2−3kb) (4.1%)
5' UTR (0.37%)
3' UTR (1.53%)
1st Exon (1.4%)
Other Exon (1.99%)
1st Intron (8.98%)
Other Intron (14.46%)
Downstream (<=300) (0.08%)
Distal Intergenic (15.57%)
6T_IRF2−2_macs3_SPMR_peaks
Numbers of filtered peaks: 27777
Promoter (<=1kb) (46.51%)
Promoter (1−2kb) (5.82%)
Promoter (2−3kb) (4.25%)
5' UTR (0.31%)
3' UTR (1.53%)
1st Exon (1.39%)
Other Exon (2.02%)
1st Intron (8.52%)
Other Intron (13.36%)
Downstream (<=300) (0.09%)
Distal Intergenic (16.21%)
C
6T_ETV6−1_macs3_SPMR
Numbers of filtered peaks: 2476
Promoter (<=1kb) (25.81%)
Promoter (1−2kb) (5.37%)
Promoter (2−3kb) (4.97%)
5' UTR (0.08%)
3' UTR (2.02%)
1st Exon (1.41%)
Other Exon (2.67%)
1st Intron (12.68%)
Other Intron (19.87%)
Downstream (<=300) (0.2%)
Distal Intergenic (24.92%)
6T_ETV6−2_macs3_SPMR
Numbers of filtered peaks: 7710
Promoter (<=1kb) (28%)
Promoter (1−2kb) (5.55%)
Promoter (2−3kb) (4.73%)
5' UTR (0.35%)
3' UTR (2.08%)
1st Exon (1.45%)
Other Exon (2.56%)
1st Intron (12.17%)
Other Intron (19.05%)
Downstream (<=300) (0.05%)
Distal Intergenic (24.01%)
