# Supplementary material for: Enhancer looping protein LDB1 modulates MYB expression in T-ALL cell lines in vitro by cooperating with master transcription factors
Source: J Exp Clin Cancer Res. 2024 Oct 9;43:283. doi: 10.1186/s13046-024-03199-1 (PMC11462673; doi:10.1186/s13046-024-03199-1)
Supplement: Supplementary file 7 — Supplementary Material 7. Supplementary Figure7. Pie chart exhibiting the DNA binding sites distribution for H3K27AC antibody in sh-NC and sh-LDB1 of 6T-CEM cells using CUT&Tag experiments. A. H3K27AC antibody/sh-NC and sh-LDB1 of 6T-CEM cells .B. After knocking down the expression level of LDB1, the enrichment of H3K27ac at the positions where LDB1 binds on the chromatin is reduced [file 13046_2024_3199_MOESM7_ESM.pdf]

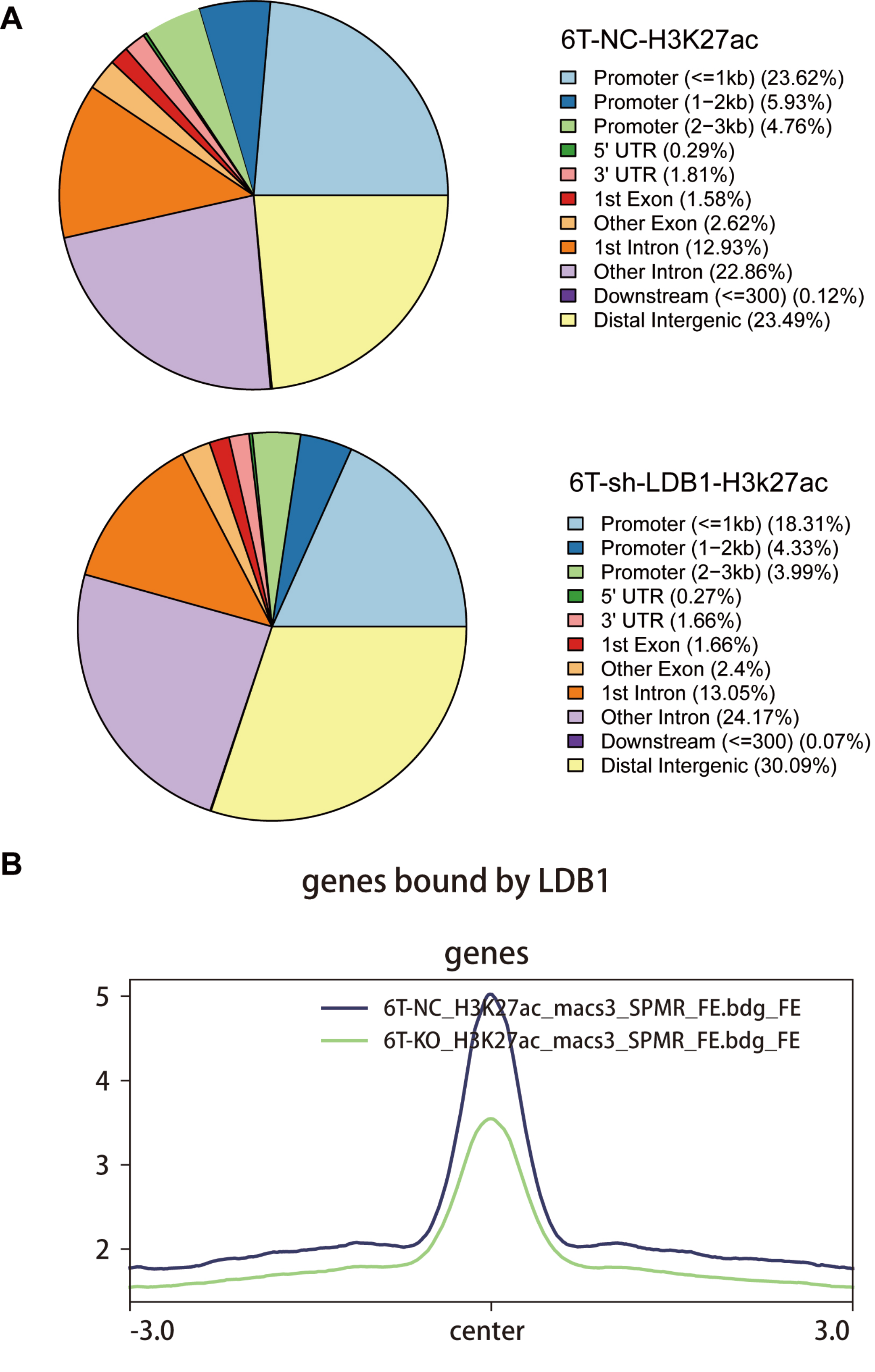
A
6T-NC-H3K27ac
Promoter (<=1kb) (23.62%)
Promoter (1−2kb) (5.93%)
Promoter (2−3kb) (4.76%)
5' UTR (0.29%)
3' UTR (1.81%)
1st Exon (1.58%)
Other Exon (2.62%)
1st Intron (12.93%)
Other Intron (22.86%)
Downstream (<=300) (0.12%)
Distal Intergenic (23.49%)
6T-sh-LDB1-H3k27ac
Promoter (<=1kb) (18.31%)
Promoter (1−2kb) (4.33%)
Promoter (2−3kb) (3.99%)
5' UTR (0.27%)
3' UTR (1.66%)
1st Exon (1.66%)
Other Exon (2.4%)
1st Intron (13.05%)
Other Intron (24.17%)
Downstream (<=300) (0.07%)
Distal Intergenic (30.09%)
B
genes bound by LDB1
genes
6T-NC_H3K27ac_macs3_SPMR_FE.bdg_FE
6T-KO_H3K27ac_macs3_SPMR_FE.bdg_FE
5
4
3
2
-3.0
center
3.0
